# From Junk DNA to Genomic Treasure: Impacts of Transposable Element DNA, RNA, and Protein in Mammalian Development and Disease

**DOI:** 10.1002/wrna.70022

**Published:** 2025-08-13

**Authors:** Ten D. Li, Katelyn Toohill, Andrew J. Modzelewski

**Affiliations:** ^1^ Department of Biomedical Sciences School of Veterinary Medicine, University of Pennsylvania Philadelphia Pennsylvania USA; ^2^ Perelman School of Medicine University of Pennsylvania Philadelphia Pennsylvania USA; ^3^ Department of Biomedical Sciences, School of Veterinary Medicine University of Pennsylvania Philadelphia Pennsylvania USA

**Keywords:** *cis*‐regulatory, development and disease, functional RNA, transposons, viral protein

## Abstract

Transposable elements (TEs) have hijacked cellular machineries to replicate and spread throughout host genomes. TEs now make up a significant portion of eukaryotic genomes and play notable roles in genomic evolution, driving both speciation and providing raw material for genetic innovation. Barbara McClintock's pioneering work on these “jumping genes” laid the foundation for modern TE research; however, her paradigm‐shifting theories in which TEs act as “controlling elements” were initially rejected due to the long‐held belief that TEs were “junk” or parasitic DNA elements. Historically, the highly repetitive nature of TEs made it challenging to both identify and investigate functions. However, recent advances in genomics have greatly accelerated our understanding of TEs. Despite their potential to cause insertional mutagenesis and disease, many transposable elements have been co‐opted by host genomes to contribute to gene regulation and development. In contrast to protein‐coding genes that typically begin their journey as DNA, are transcribed into RNA, and reach their ultimate functional form as proteins, TEs can function as *cis*‐regulatory DNA, functional RNA, and in rare cases, domesticated proteins and fusion events between TE and host genes. Driven by rapidly advancing technologies, the roles of TEs in both development and disease are being uncovered faster than ever, making current and future work an exciting continuation of Barbara McClintock's groundbreaking legacy.

## Graphical abstract

Transposable elements shape development and disease at every level of the central dogma by functioning as regulatory DNA, functional RNA, and, in some cases, protein, thus transforming our view of them from junk to essential genomic players.

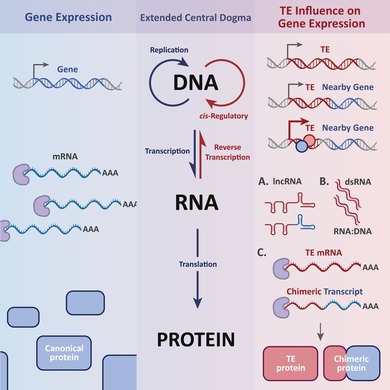

**Abbreviations**
ALS: Amyotrophic Lateral Sclerosis
cDNA: complementary DNA
CRISPR: clustered regularly interspaced short palindromic repeats
dsRNA: double‐stranded RNA
Env: envelope
ERV: endogenous retrovirus
ESC: embryonic stem cell
Gag: group specific antigen
H3K27ac: Histone 3 Lysine 27 Acetylation
H3K27me3: Histone 3 Lysine 27 Trimethylation
H3K4me1: Histone 3 Lysine 4 Monomethylation
H3K4me3: Histone 3 Lysine 4 Trimethylation
H3K9me3: Histone 3 Lysine 9 Trimethylation
HERV: human endogenous retrovirus
IAP: intracisternal A‐type particle
ICM: inner cell mass
iPSC: induced pluripotent stem cell
LINE: long interspersed nuclear element
lnc‐EPAV: ERV‐derived lncRNA positively regulates antiviral responses
lncRNA: long non‐coding RNA
LTR: long terminal repeat
MER: Mammalian‐wide interspersed repeat
MERV‐L: mouse endogenous retrovirus‐L
ncRNA: noncoding RNA
OCT4: Octamer 4
ORF: open reading frame
Pol: polyprotein
Pro: protease
RNP: RiboNucleo protein
SINE: short interspersed nuclear element
SOX: sex determining region Y Box
TBXT: T‐box transcription factor T
T‐Cell: thymus cell
TDP‐43: transactive response DNA binding protein 43 kDa
TE: transposable element
TRIM28: tripartite motif‐containing 28
UTR: untranslated region

## 1 Introduction

Transposable Elements (TEs) hijack host cellular machineries to replicate and spread in host genomes and currently contribute to a significant portion of eukaryotic genomic material (Biémont 2010). Barbara McClintock's pioneering genetics work in the 1940s postulated that these “jumping genes” could also act as “controlling elements” to directly participate in the development and differentiation of host organisms. However, this highly controversial idea would not be appreciated until many decades later (Comfort 2001) as confirmation of Barbara McClintock's theory was limited by the technologies of their time. In fact, McClintock's 1983 Nobel Prize was awarded for the description of genomic rearrangements during transposition and not for the role of TEs in developmental gene regulation that was originally proposed (Comfort 2001). Validation of this hypothesis required technical innovations that eventually led to the first draft of the human genome in 2001, which was finally completed by the Telomere to Telomere Consortium in 2022 (Nurk et al. 2022). This enabled a comprehensive understanding of human genomics and provided the tools and evidence to support a role for TEs in development and disease.

**FIGURE 1:**
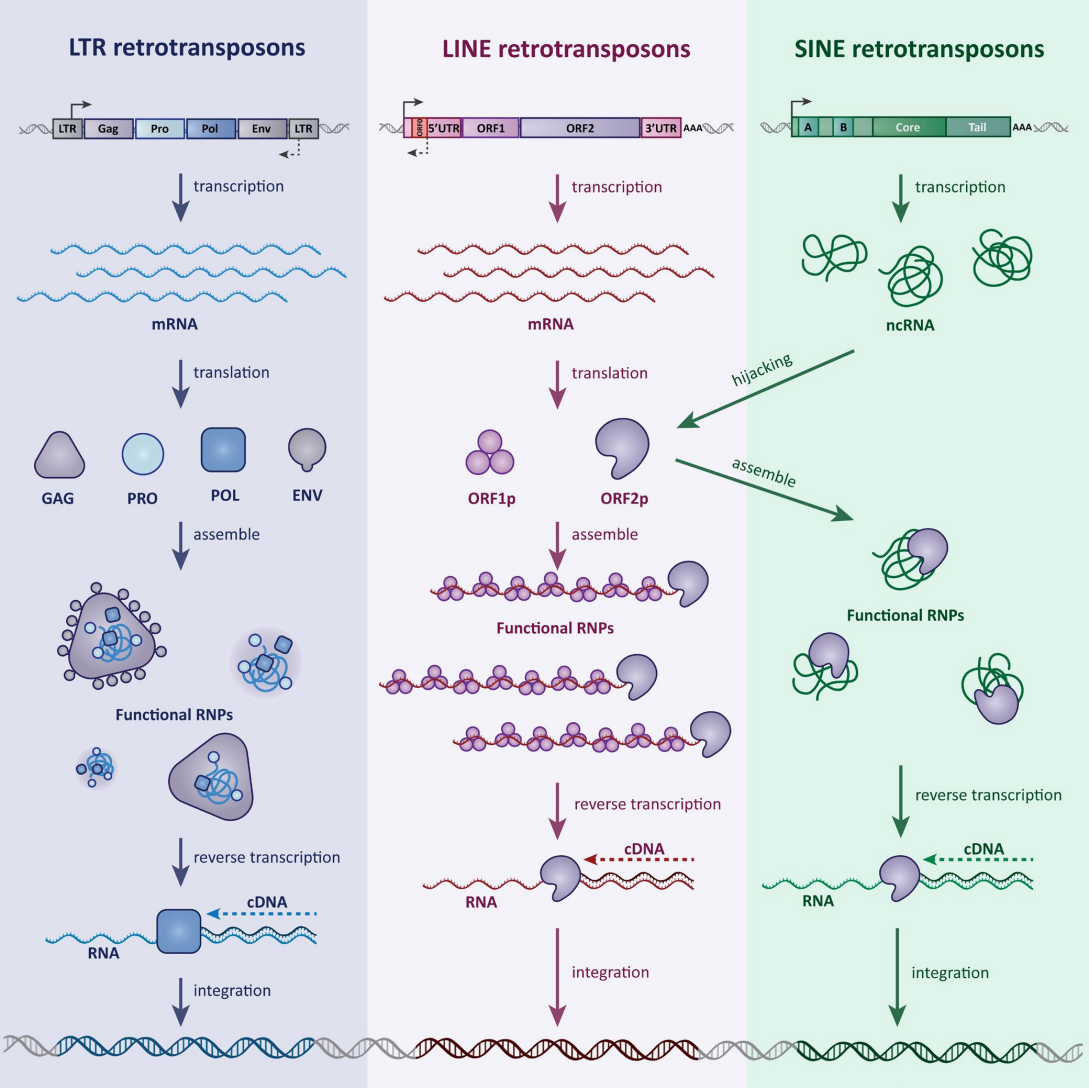
Mechanistic characterization of retrotransposons and sources of functional DNA, RNA, and Protein. The life cycle of all LTR, LINE, and SINE types of retrotransposons follows the standard pipeline of DNA, RNA, and protein components for successful retrotransposition. Once transcribed and translated, Retrotransposon RNAs and proteins form ribonucleoproteins (RNPs) as functional retrotransposition‐competent units. Retrotransposons provide a considerable number of genetic innovations to mammalian genomes, with many examples resembling aspects of ancestral retrotransposition. The three major classes of retrotransposons are largely characterized at the sequence level but also by mode of retrotransposition, which involves an RNA intermediate prior to reintegration into the genome. The long terminal repeat (LTR) class (left), in which Endogenous Retroviruses (ERVs) are a major component of this category, shares many details with currently active and infectious retroviruses. Most LTR retrotransposons code for four open reading frames that are further processed into smaller peptides, but the Envelope (Env) protein is frequently lost early in colonization. While some LTRs can assemble seemingly intact and complete viral particles, this is not necessary for reinsertion into the genome. LINE elements (center) have three open reading frames (ORFs), although only ORF1 and ORF2 functions have been characterized. Long interspersed nuclear elements (LINEs) do not resemble active viral elements, and their origins are unclear. Short interspersed nuclear elements (SINEs) elements (right) are not protein‐coding but instead utilize LINE element machinery to colonize the genome. All stages of retrotransposition found in all three classes provide an opportunity for host genome domestication.

The diversity of mammalian TEs is staggering but can be simplified when classified by mode of transposition (Figure 1). DNA transposons, first studied by Barbara McClintock, make up 3%–4% of the human genome. Although DNA transposons are no longer active in mammalian genomes, they once mobilized through excision and insertion of DNA through a so‐called “cut & paste” mechanism (Wells and Feschotte 2020). In contrast, retrotransposons replicate using a complex “copy & paste” mechanism in which an RNA intermediate is generated and is then reverse transcribed into complementary DNA (cDNA) preceding integration (Figure 1). Retrotransposons are further divided by the presence or absence of identical “Long Terminal Repeats”, or LTRs, that flank their internal protein‐coding regions. Non‐LTR retrotransposons include Long Interspersed Nuclear Elements (LINEs, 21% of the human genome (Martin 2018; Zhang et al. 2020)) and the non‐autonomous Short Interspersed Nuclear Elements (SINEs, 13% of the human genome (Hoyt et al. 2022; Zhang et al. 2021)). While the origins of LINE and SINE elements are unclear, the majority of LTR retrotransposons can be traced back to ancient retroviral infections and are frequently referred to as Endogenous Retroviruses, or ERVs, that now make up 9% of the human genome. During colonization and retrotransposition, both LINEs and ERVs are transcribed much like protein‐coding genes, where their DNA is first transcribed into messenger RNA and further translated into protein (Figure 1). SINE elements, on the other hand, are non‐autonomous and rely on LINE machinery to support their propagation, which involves hijacking of newly translated LINE proteins (Figure 1). Unlike protein‐coding genes, at this stage retrotransposon proteins then directly interact with their own RNA (or hijack LINE protein in the case of SINE RNA), to form ribonucleoproteins (RNPs). In all of these cases, retrotransposon RNPs have been determined to have multiple functions to support propagation, but the most unique aspect is the ability of these RNPs to reverse transcribe their cognate RNA into complementary DNA, which is a critical step in the integration of a brand‐new *copy* of a retrotransposon prior to *pasting* it into the genome (Figure 1). The RNP and newly made DNA go onto generate potentially mutagenic cuts in the genome and hijack cellular machine to efficiently integrate the new DNA (Figure 1). Over millions of years, TE colonization of genomes has been extremely successful and as such the precise TE repertoire between species is unique, thus forcing each host genome to adapt to TEs uniquely present in their respective genomes.

**FIGURE 2:**
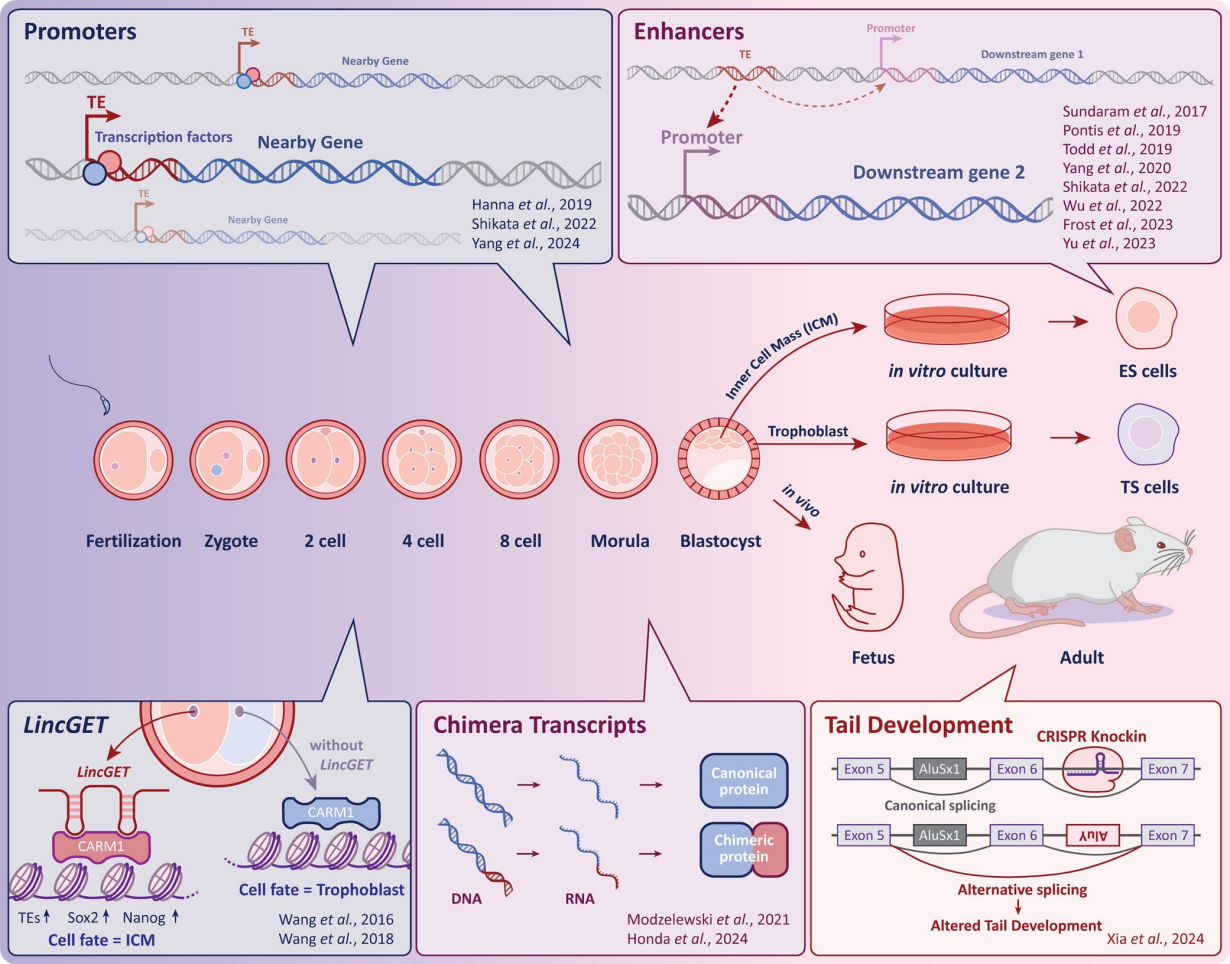
Functional impact of transposable element DNA and RNA on early development. The major developmental stages from fertilization to adulthood are shown (center). At the blastocyst stage, the inner cell mass (ICM) could become mouse embryonic stem cells (ESCs) under *in vitro* culture, and *in vitro* culturing of trophoblasts results in establishing trophoblast stem cells. Upper panels show TE functional DNA mechanisms of enhancer and promoter activity in various developmental stages. (From left to right of upper panels) Shikata et al. and Yang et al. both found LTR transposons act as *cis*‐regulatory elements during mouse zygotic genome activation where LTR elements show promoter activity that would impact pre‐implantation embryogenesis (Shikata et al. 2022; Yang et al. 2024). A study showed a causative role for MERV‐L in 2‐Cell Like Cells in which MERV‐L displays enhancer activity for downstream genes in mouse ESCs (Yang et al. 2020). Various groups reported that CRISPR deletion of TEs results in global gene expression change in ESCs or trophoblast stem cells, suggesting TEs as functional *cis*‐regulatory elements in stem cells as well (Hanna et al. 2019; Pontis et al. 2019; Shikata et al. 2022; Todd et al. 2019; Wu et al. 2022; Yu et al. 2023). Lower panels show representative studies in recent years of TE RNA based mechanisms. (From left to right of lower panels) Wang et al. revealed that a lncRNA associated with Gene Expression and Transcription (*lincGET*) asymmetrically expressed in one of the 2‐cell blastomeres and promotes it to develop to ICM (Wang et al. 2016, 2018). TE‐fused chimeric transcript‐derived chimeric protein products showed distinct functions in early development. Depletion of chimeric transcripts leads to severe misregulation during embryogenesis, resulting in lethality (Honda et al. 2024; Modzelewski et al. 2021). An Alu element insertion causes alternative mRNA splicing, and the exon‐skipped transcript that results in altered tail (Xia et al. 2024).

**FIGURE 3:**
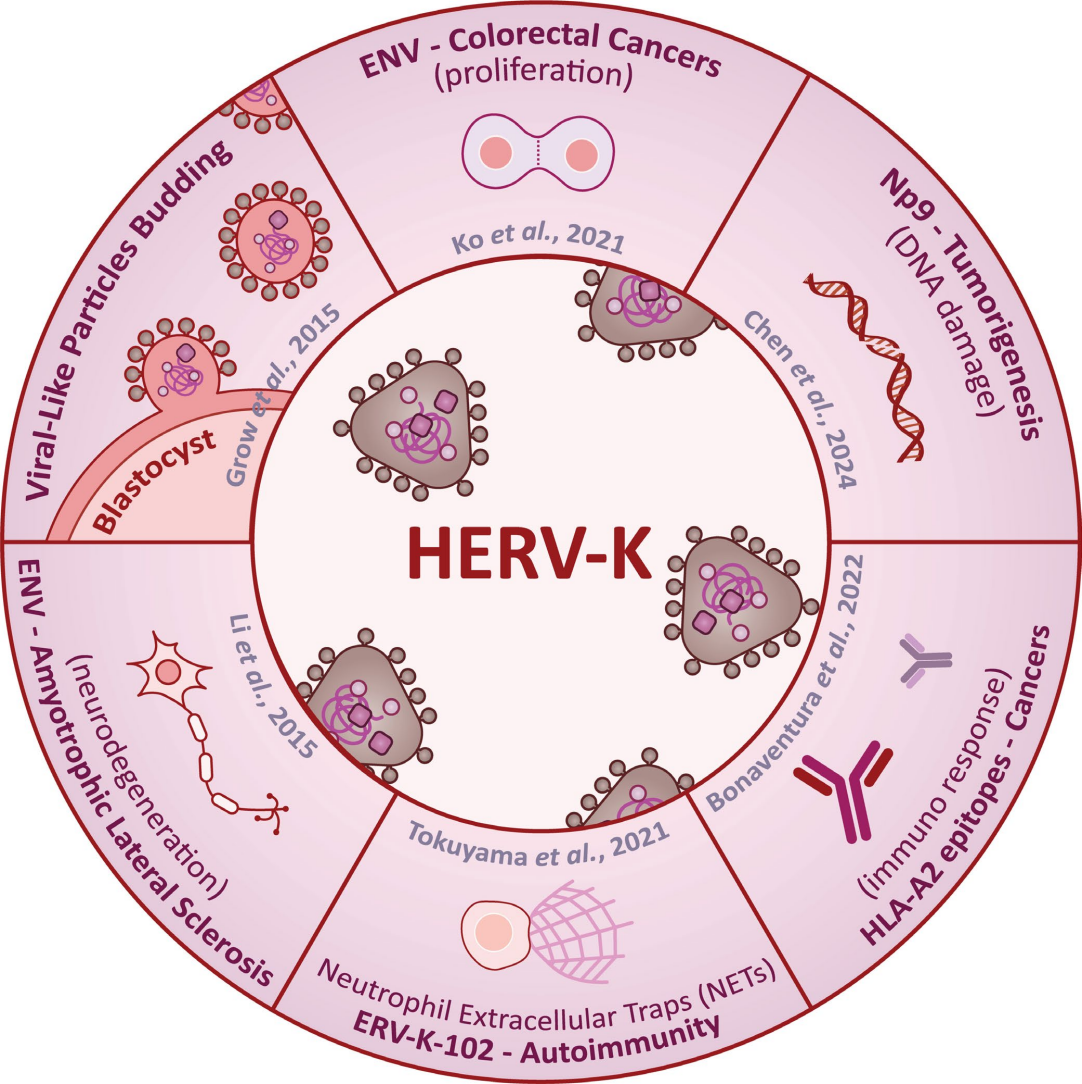
HERV‐K proteins contribute to human disease. Human endogenous retrovirus K, or HERV‐K, is among the most active human ERVs. While rare compared to solo‐LTRs, structurally intact HERV‐K loci exist, from which proteins such as groups specific antigen (Gag), polyprotein (Pol), Env, and NP9 are translated in specific diseased biological contexts. HERV‐K Env expression in colorectal cancer promotes the expression of Nuclear Protein 1, contributing to cell proliferation, tumor growth, and migration through a related pathway (Ko et al. 2021). HERV‐K NP9 protein expression is associated with upregulates markers of DNA damage, leading to increased levels of DNA mutagenesis (Chen et al. 2024). HERV‐K peptides are translated in solid tumor cancers, serving as human leukocyte antigens (HLA‐A2) epitopes and inducing immunogenic responses (Bonaventura et al. 2022). Systemic Lupus Erythematosus patients develop autoantibodies against the Env of HERV‐K102, inducing neutrophil activation and Neutrophil Extracellular Traps (NETs) formation (Tokuyama et al. 2021). HERV‐K Env is expressed in Amyotrophic Lateral Sclerosis (ALS) patient subpopulations, causing neurite dysfunction in cultured human neurons and progressive motor dysfunction in HERV‐K Envelope transgenic mice (Li et al. 2015). Specific functional roles for HERV‐K proteins in development remain unelucidated; however, evidence suggests HERV‐K Gag and virus‐like particles may serve roles in pre‐implantation embryogenesis (Grow et al. 2015).

When first colonizing, TEs represent an inherent danger to the host genome (Figure 1), as their transposition can result in the insertional mutagenesis of essential genes. Therefore, various epigenetic host defense mechanisms have arisen to safeguard genome integrity during transposition (Inoue et al. 2017). Likewise, there are clear host‐driven selective pressure to reduce TE evolution. These suppression mechanisms have largely been successful such that the overwhelming majority of current TEs are “fossils” or fragments of their original sequences. A significant subset of TEs nonetheless maintain a presence in the genome that are recognized by host transcriptional machinery (Tables 1 and 2) and can even be transcribed (Table 3) and translated (Table 4). An increasing number recent reports suggest that these TEs can sometimes be repurposed by host genomes (Figure 1). As TE insertions are largely species‐specific, host species respond to their presence in species‐specific manners. As a result, these fossils are a potential source of species‐specific evolutionary adaptions (Figure 2). On the slow path to becoming neutralized, silenced TEs gradually degrade and no longer pose threats to the genome, with each TE category decaying in unique ways. LINE elements propagate through reverse transcription, after which they integrate throughout the genome. Reverse transcription is an inefficient process that often leads to insertions being significantly truncated at their 5′ ends, reducing their 5′ untranslated regions (UTRs) and thus their own regulatory capacity (Ghanim et al. 2025; Kazazian and Moran 2017; Szak et al. 2002). In fact, only 80–100 evolutionarily young copies of human LINE‐1 elements appear to be full‐length and retrotransposition competent (Ebert et al. 2021). Additionally, a small number of the SINE related non‐autonomous AluY and the human and great ape‐specific SVA or “SINE–variable number tandem repeat‐*Alu*” sequences are retrotransposition competent. As these retrotransposons rely on LINE machinery for mobilization (Burns 2017; Thawani et al. 2024) they tend to become extinct in parallel to LINE self‐inactivation. Like LINEs and SINEs, ERVs are inactivated through the slow accumulation of mutations. As a result, ERVs lose protein‐coding capacity. However, their flanking LTRs, which are identical to each other, have the tendency to recombine with each other, leaving a solitary LTR where a full ERV once was. Most ERVs exist as these solitary LTRs and are no longer believed to mobilize or pose a genomic threat in the human genome in this state (Wells and Feschotte 2020). However, these solitary LTRs are densely packed with regulatory information and transcription factor binding sites (Table 1) which are scattered all throughout current mammalian genomes. This arrangement carries the potential to influence nearby gene expression under certain cellular contexts (Table 2) in which TE silencing is relaxed (Figure 2) or misregulated (Figure 3). Collectively, universal TE colonization has left metazoan genomes saturated with the remains of ancient TE fossils, of which a vanishingly small number pose any major threat to the host but instead have left millions of insertions with the potential to be utilized by the host genome.

**TABLE 1:** TEs as a source of transcription factor binding sites.

Major TE	Species	Feature	Frequency	References
Various	Human	Enhancers	45.0%	(Simonti et al. 2017)
Various	Mouse	Enhancers	37.0%	(Simonti et al. 2017)
Various	Human	DNA methylation at CpG	50.0%	(Pehrsson et al. 2019)
Various	Human	CTCF	22.5%	(Sundaram et al. 2014)
ERV‐L/ERV1	Human	CTCF	11.1%	(Kunarso et al. 2010)
SINE (B2)	Mouse	CTCF	33.8%	(Bourque et al. 2008)
SINE (B2)	Mouse	CTCF	26.0%	(Xie et al. 2010)
SINE (B2)	Mouse	CTCF	28.3%	(Kunarso et al. 2010)
Various	Mouse	CTCF	40.0%	(Sundaram et al. 2014)
LTR	Mouse	OCT3/4	20.2%	(Sundaram et al. 2017)
ERV‐K (ERV2)	Mouse	OCT3/4	7.2%	(Kunarso et al. 2010)
ERV1	Human	OCT3/4	20.9%	(Kunarso et al. 2010)
ERV‐K (ERV2)	Mouse	OCT3/4 + SOX2	23.8%	(Bourque et al. 2008)
ERV‐K (ERV2)	Mouse	SOX2	16.0%	(Xie et al. 2010)
LTR	Mouse	SOX2	26.9%	(Sundaram et al. 2017)
ERV‐K (ERV2)	Mouse	NANOG	17.1%	(Kunarso et al. 2010)
ERV1	Human	NANOG	14.6%	(Kunarso et al. 2010)
ERV‐K (ERV2)	Mouse	NANOG	16.0%	(Xie et al. 2010)
LTR	Mouse	NANOG	28.5%	(Sundaram et al. 2017)
ERV‐K (ERV2)	Mouse	EP300	12.0%	(Xie et al. 2010)
Various	Mouse	EP300	19.6%	(Sundaram et al. 2014)
Various	Human	EP300	27.3%	(Sundaram et al. 2014)
ERV1	Mouse	p53	39.6%	(Bourque et al. 2008)
HER‐V/LTR	Human	p53	30.0%	(Wang et al. 2007)
Various	Human	MYC	35.2%	(Jiang and Upton 2019)
Various	Mouse	MYC	20.4%	(Sundaram et al. 2014)
SINE	Mouse	ESRRB	26.4%	(Sundaram et al. 2017)
SINE (B4)	Mouse	ESRRB	17.0%	(Xie et al. 2010)
Various	Human	GATA1	25.7%	(Sundaram et al. 2014)
Various	Mouse	GATA1	21.8%	(Sundaram et al. 2014)
Various	Mouse	RAD21	32.9%	(Sundaram et al. 2014)
Various	Human	RAD21	19.2%	(Sundaram et al. 2014)
LTR	Mouse	KLF4	13.3%	(Sundaram et al. 2017)
MIR	Mouse	ESR1	19.8%	(Bourque et al. 2008)
Various	Human	C/EBPb	54.6%	(Jiang and Upton 2019)
Various	Human	E2F1	36.2%	(Jiang and Upton 2019)
Various	Human	255 TFs combined	17.0%	(Nikitin et al. 2018)
HER‐V/LTR	Human	97 TFs combined	12.0%	(Ito et al. 2017)
L1	Human	97 TFs combined	15.0%	(Ito et al. 2017)
SINE	Human	97 TFs combined	16.0%	(Ito et al. 2017)
DNA	Human	97 TFs combined	6.0%	(Ito et al. 2017)
Various	Human	26 TFs combined	19.0%	(Sundaram et al. 2014)
Various	Mouse	26 TFs combined	20.0%	(Sundaram et al. 2014)
Various	29 Mammals	Conserved Non Exonic Elements	19.6%	(Lowe and Haussler 2012)

*Note:* TEs serve as a major source of transcription factor binding sites. Major TE categories are identified from each reference and further broken down into species. Feature refers to what potential or validated transcription factor binding site the TE provides in its sequence. Frequency refers to the percent of the specific transcription factor binding site that the major TE contributes to the respective genome.

**TABLE 2:** Recent studies showing TEs as c*is*‐regulatory elements.

TE	Species	Biological context	Function	Functional study	References
LTR5HS	Human	Human embryonal carcinoma NCCIT cells	Enhancer	CARGO CRISPR activation/CRISPR inhibition	(Fuentes et al. 2018)
RLTRs, RLTR13D6	Mouse	Embryonic stem cells, Trophoblast stem cells	Enhancer	CRISPR deletion, CRISPR inhibition of RLTR13D6 elements	(Todd et al. 2019)
AluJb	Human	Lung cancer cell lines	Promoter	CRISPR deletion	(Jang et al. 2019)
LTR5H, LTR7B, LTR7Y	Human	Embryonic stem cells	Enhancer	CRISPR inhibition	(Pontis et al. 2019)
ERV‐K LTRs	Mouse	Extra‐embryonic lineages	Promoter	CRISPR deletion	(Hanna et al. 2019)
RLTR10B	Mouse	Spermatogenesis	Enhancer	Luciferase assays, CRISPR activation, CRISPR deletion	(Sakashita et al. 2020)
LTR2B, LTR2C, LTR5B, LTR5HS, LTR12C and LTR13A	Human	Acute myeloid leukemia	Enhancer	CRISPR inhibition, CRISPR deletion	(Deniz et al. 2020)
LTR12C	Human	HIV‐1 infection of CD4+ T‐cells	Promoter	Luciferase assays, fluorescent reporter constructs	(Srinivasachar Badarinarayan et al. 2020)
RSINE1	Mouse	Liver (Hepa 1–6 cell lines)	Enhancer	Luciferase assays	(Judd et al. 2021)
MERV‐L	Mouse	Embryonic stem cells, pre‐implantation embryogenesis	Enhancer	CRISPR activation	(Yang et al. 2020)
LTR5H	Human	Primordial germ cells	Enhancer	CRISPR inhibition	(Xiang et al. 2022)
LTR6B	Human	Definitive endoderm cells	Enhancer	CRISPR deletion	(Wu et al. 2022)
RLTR45	Mouse	Pre‐implantation embryogenesis (Somatic cell nuclear transfer)	Enhancer	CRISPR deletion	(Shikata et al. 2022)
LTR1/LTR1a	Mouse	Neural progenitor cells (NPCs)	Enhancer	Fluorescent reporter constructs	(Enriquez‐Gasca et al. 2023)
MER41B, LTR10A	Human	Cytotrophoblast‐like trophoblast stem cells	Enhancer	CRISPR deletion	(Frost et al. 2023)
B2_Mm2	Mouse	Bone‐marrow‐derived macrophages (BMDMs), Macrophage‐like cells	Enhancer	CRISPR deletion	(Horton et al. 2023)
MER11B	Human	Colon cancer (GP5d cells)	Enhancer	CRISPR deletion	(Karttunen et al. 2023)
MER50	Human	Trophoblast stem cells	Enhancer	CRISPR deletion	(Yu et al. 2023)
Mt2_Mm	Mouse	Pre‐implantation embryogenesis	Enhancer/promoter	CRISPR inhibition	(Yang et al. 2024)
LTR12C	Human	*In vitro* human cell line (HEK293T)	Enhancer/promoter	CRISPR activation	(Ohtani et al. 2024)
LTR10	Human	Colorectal cancer (HCT116 cells and primary tumor cells)	Enhancer	CRISPR inhibition, CRISPR deletion	(Ivancevic et al. 2024)
LTR8B	Human	Cancer (HT1080 fibrosarcoma)	Promoter	CRISPR inhibition	(Dziulko et al. 2024)

*Note:* TEs have been reported to function as *cis*‐regulatory elements, non‐coding DNA elements that regulate the transcription of nearby genes, in context‐specific developmental and diseased conditions. TEs are mostly commonly reported to function as enhancers; however, they have been described to serve other functions such as acting as promoters. Functional study refers to the type of experiment conducted to help determined the function of the TE. This table provides reports of recent functional studies of *cis*‐regulatory TEs.

**TABLE 3:** Recent studies showing TEs as functional RNA.

TE	Species	Biological context	Function	Functional study	References
Alu	Human	*In vitro*	Splicing	Minigene‐splicing assay	(Payer et al. 2019)
GLN, MERV‐K, and MERV‐L	Mouse	Pre‐implantation embryogenesis	lncRNA (*LincGET*)	Knockdown, overexpression	(Wang et al. 2018)
ERV1	Mouse	Macrophages	LncRNA (*ERV‐derived lncRNA positively regulates antiviral responses, lnc‐EPAV*)	Overexpression, knockdown, knockout	(Zhou et al. 2019)
B2, Alu	Mouse/Human	T‐cell activation, thermal and endoplasmic reticulum stress	Ribozyme	*In vitro* cleavage assays, cellular stress tests	(Hernandez et al. 2020)
ERVs (various)	Human	Influenza virus infection	dsRNA	Knockout of MAVS and cGAS‐STING	(Schmidt et al. 2019)
LTR70	Human	Triple negative breast cancer	lncRNA (*TROJAN*)	Knockdown, knockout, overexpression, mouse *in vivo* metastasis/xenograft studies	(Jin et al. 2019)
MER41	Human	Fetal cardiomyocytes	lncRNA (*BANCR*)	Knockdown, overexpression, knockout	(Wilson et al. 2020)
ERVs (various)	Mouse	Breast and lung cancer	dsRNA	DsRNA analogue supplementation; tumoral dsRNA pulldown	(Tavora et al. 2020)
B2	Mouse	Amyloid beta pathology (hippocampus)	ncRNA	Knockdown	(Cheng et al. 2020)
ERVs (various)	Mouse	*Staphylococcus epidermidis* colonization (keratinocytes)	cDNA	Antiretroviral treatment	(Lima‐Junior et al. 2021)
L1, SINEs	Blind Mole Rat	Concerted cell death	RNA/DNA hybrids	RNase H treatment, cGAS knockdown, murine xenograft experiments	(Zhao et al. 2021)
L1	Human	T‐cell quiescence	Chimeric transcripts	Pan L1 knockdown	(Marasca et al. 2022)
IAP	Mouse	Embryonic stem cells, pre‐implantation embryogenesis	RNA scaffold	Knockdown	(Asimi et al. 2022)
MERV‐L	Mouse	Pre‐implantation embryogenesis	Transcription‐associated	Knockdown, CRISPR inhibition	(Sakashita et al. 2023)
LTR7C	Human	Trophoblast stem cells	lncRNA *(Urothelial Cancer Associated 1 (UCA1))*	Overexpression, knockdown	(Kong et al. 2024)
SVP	Human	Skin pigmentation	Splicing	Splicing construct	(Kamitaki et al. 2024)
MLT2B3	Human	Hepatocellular carcinoma	lncRNA (LINC01446)	Overexpression, knockdown	(Wu et al. 2024)
Alu	Human	Embryogenesis	Splicing	CRISPR deletion, humanized mouse model	(Xia et al. 2024)
RNLTR12	Rat	Oligodendrocytes and oligodendrocyte progenitor cells	lncRNA (*RetroMyelin*)	Knockdown	(Ghosh et al. 2024)

*Note:* TEs have been reported to function as non‐coding RNAs (ncRNA) and long non‐coding RNA (lncRNA) elements that can interact with other RNAs and proteins to alter biological pathways in context‐specific developmental and diseased conditions. Functional study refers to the type of experiment conducted to help determined the function of the TE. This table provides reports of recent functional studies of TE‐derived RNAs.

**TABLE 4:** Recent studies showing TEs as functional protein.

TE	Species	Transposon protein(s)	Biological context	Proposed function	References
AluJb	Human	Chimeric protein (AluJb‐LIN28B)	Lung cancer cell lines	Represses let‐7 miRNAs and promotes cell proliferation, migration, and tumor formation; similar function to the canonical isoform	(Jang et al. 2019)
HML‐2	Human	Env	Pluripotent stem cells	Signals via binding to CD98HC; maintains stemness	(Wang et al. 2020)
HERV‐W	Human	Env	Hippocampus (glial and hippocampal cells)	HERV‐W Env accelerates synaptic maturation and alters glutamatergic signaling	(Johansson et al. 2020)
MLV	Mouse	Env	Immune cells (B‐cells, T‐cells, primary lymphocytes)	Antibody ligation to MLV Envelope proteins on the plasma membrane leads to internalization and initiate signaling cascades	(Panova et al. 2020)
HERVs (various)	Human	Peptides	T‐cells (healthy and myeloid malignancies)	T‐cells recognize HERV peptides in myeloid malignancies, highlighting their potential to be therapeutically targeted	(Saini et al. 2020)
Unspecified	Mouse	Reverse transcriptase	Embryonic stem cells	Protect against viral infection through reverse transcription of viral RNA into viral complementary DNA and then the formation of viral RNA/DNA hybrids, leading to destruction of viral RNA by RNase H1	(Wu et al. 2021)
HERV‐K‐102	Human	Env	Systemic lupus erythematosus (SLE), Neutrophils	SLE patients form autoantibodies against HERV‐K‐102 which can activate neutrophils when in a complex with SLE IgG	(Tokuyama et al. 2021)
HERV‐W	Human	Env (Syncytin‐1)	Epstein–Barr virus and Kaposi's sarcoma‐associated herpesvirus infections (B‐cells)	Aids Epstein–Barr virus and Kaposi's sarcoma‐associated herpesvirus lytic replication activation from latency; potentially promotes replication	(Frey et al. 2021)
HERV‐K	Human	Env	Colorectal cancer cell lines	Promotes expression of NUPR1; promotes proliferation, tumor growth, and migration through a NUPR1 related pathway	(Ko et al. 2021)
MT2B2	Mouse	*Cdk2ap1* ^*MT2B2*^	Pre‐implantation embryogenesis	Promotes cell cycle progression and proliferation; opposing function to canonical *Cdk2ap1*	(Modzelewski et al. 2021)
HERVs (various)	Human	Peptides	Cancer (solid tumors), CD8+ T‐cells	ERV HLA‐A2 epitopes are immunogenic and induce T‐cells clones which kill tumor cells presenting HERV epitopes	(Bonaventura et al. 2022)
HERV‐K	Human	Retrovirus‐like particles	Aging/senescence (Hutchinson‐Gilford progeria syndrome human mesenchymal progenitor cells)	Extracellular HERV‐K retroviral‐like particles spread senescence from old to young cells	(Liu et al. 2023)
Ty3/Gypsy	Human	Gag‐Pol (Paternally expressed gene 10 (PEG10))	Amyotrophic Lateral Sclerosis (ALS) spinal cord tissue	Contributes to ALS through altering expression of axon remodeling genes; regulated by ALS‐causing Ubiquilin 2 (UBQLN2)	(Black et al. 2023)
Ty3/Gypsy	Mouse	Gag‐Pol (Paternally expressed gene 10 (PEG10))	Placental Function	Domesticated retrotransposon crucial for trophoblast cell proliferation and placental formation, especially in the labyrinth and spongiotrophoblast layers.	(Ono et al. 2006)
HERV‐H	Human	Chimeric protein (HERV‐H Calbindin)	Lung squamous cell carcinoma	Prevents senescence during cancer initiation and prevents senescence‐associated secretory phenotype (SASP)	(Attig et al. 2023)
MLV, HERV‐K	Mouse/Human	Env	Lung adenocarcinoma (LUAD)	Expressed in mouse and human LUAD; target of anti‐tumor antibodies	(Ng et al. 2023)
MERV‐L	Mouse	Gag	Pre‐implantation embryogenesis, embryonic stem cells, 2‐cell‐like cells	Protects OCT4 and SOX2 from degradation by competitively binding to URI (unconventional prefoldin RPB5 interactor)	(de la Rosa et al. 2024)
ERVH48‐1	Human	Env fragment	Mesoderm and cardiomyocyte differentiation (pluripotent stem cells)	Inhibits secreted frizzled‐related protein 2 to regulate WNT/β‐catenin signaling; necessary for proper cardiomyocyte and mesoderm differentiation	(Zhang et al. 2024)
MT2B2	Mouse	*Prmt6* ^*MT2B2*^	Pre‐implantation embryogenesis	Promotes cell proliferation and blastomere differentiation into epiblast cells	(Honda et al. 2024)
HERV‐K	Human	NP9	*In vitro* cell lines (HEK293A), Kaposi's Sarcoma‐associated herpesvirus infected cells	Induces/enhances DNA damage by upregulating γH2AX; decreases expression of latency‐associated nuclear antigen	(Chen et al. 2024)

*Note:* While most TE loci are no longer capable of transposition due to mutagenesis and silencing, some TEs retain the capability to transcribe and translate functional TE proteins. This table provides reports of recent functional studies of TE‐derived proteins.

While approximately 2% of the human genome contains protein‐coding genes, at least 50% of the genome appears to originate from TEs, outnumbering protein‐coding genes about 200 to 1 (Hoyt et al. 2022; Lander et al. 2001). Despite obvious threats to genome integrity, TEs and their fragments maintain a strong presence in host genomes, suggesting they may not be completely detrimental to host fitness (Doolittle and Sapienza 1980). What might TEs offer in exchange for delayed repulsion from the genome?

One possible answer comes from comparing gene and TE regions across species. Only 4% of mammalian genomes are under obvious evolutionary constraint, which is mostly at protein‐coding regions (Bourque et al. 2018; Lindblad‐Toh et al. 2011; Venuto and Bourque 2018). In stark contrast, sequence conservation of TEs is low, often even species‐specific. This suggests that during evolution, hosts were forced to adapt to their distinct TE composition with unique species‐specific co‐evolutionary consequences. In support, analysis of the frequency and locations of over two dozen transcription factors in mice and humans revealed a surprisingly high number of transcription factor binding sites were found within TE sequences. This finding proposes that TEs often provide regulatory options to nearby genes but not necessarily in fixed positions when compared between species. As TE integrations are thought to be largely random, the placement of TE‐derived transcription factor binding sites appears to be unique to each species (Bourque et al. 2008; Sundaram et al. 2014). As such, their presence may have functional consequences on nearby protein‐coding genes, arguing that TEs may play an underappreciated role in *cis*‐regulatory gene expression (Tables 1 and 2). Another possible answer involves TE RNA, which originally adopted specific conformations to be efficiently recognized by cognate proteins to form RNPs (Figure 1). These features may have persisted in TEs still capable of being transcribed or reactivated, which is a well‐documented phenomenon in various developmental and disease contexts (Figures 2 and 3, Table 3). Finally, individual TE proteins have the interesting reputation of having developed multiple and distinct functions and enzymatic activities to support their own propagation, a feat best demonstrated by LINE machinery (Baldwin et al. 2024; Havecker et al. 2004). While exceedingly rare, there are examples of TE fragments that are still capable of producing proteins. These are often similar but not identical to their originally encoded versions or result from ancient fusion events that provide novel functions to protein‐coding genes, where examples of each emerged in recent exciting literature (Figures 2 and 3, Table 4). Therefore, many of the original steps from the TE life cycle and mechanisms used for selfish propagation have potentially been co‐opted for function to the benefit of the host.

Here we provide a review of recent reports in which TEs were evidenced to have a functional impact on mammalian development and disease in the context of *cis‐*regulatory DNA elements (Table 1 and Table 2), functional RNAs (Table 3) and in some cases, TE proteins or TE‐Gene fusion proteins (Table 4). We focus on the functional characterization of TEs, technological improvements allowing for novel discovery, and key challenges and opportunities in this exciting new field of study. For clarity and ease of navigation, we provide the following outline to guide readers through the structure of this review.

Navigational outline: TE function across species and contexts.

**Table:** 

Species or context	DNA function	RNA function	Protein function
Human	2.1 Evidence of TE DNA activity in human	3.1 Evidence of functional TE RNA in human	4.1 Evidence of functional TE protein in human and mouse development
Mouse	2.2 Evidence of TE DNA activity in mouse	3.2 Evidence of functional TE RNA in mouse	
Disease	2.3 Evidence of TE DNA activity in disease	3.1 Evidence of functional TE RNA in disease	4.2 Evidence of functional TE protein in disease

## 2 Transposons as an Overlooked Source of *Cis*‐Regulatory DNA

*Cis*‐regulatory elements are non‐coding regions in DNA that influence neighboring gene expression at the transcriptional level, often with cell type‐specific function (De Santa et al. 2010; Kim et al. 2010). With nearly four million annotated TEs in the human genome compared to approximately 20,000 protein‐coding genes, TEs provide a considerable amount of raw material for genomic innovation (Balachandran et al. 2022; Mills et al. 2007; Salzberg 2018) and are an abundant source of potential regulatory information (Raney et al. 2024). In comparison to the larger genomic footprint of regulatory regions of protein‐coding genes (hundreds to multiple thousands of base pairs), LINEs, SINEs, and LTRs harbor more densely packed regulatory information in the form of transcription factor binding sites within their relatively smaller UTRs and LTRs (hundreds of base pairs), respectively (Lowe and Haussler 2012; Polak and Domany 2006). The origin of these transcription factor binding sites is likely evolutionary responses to host‐evolved defense mechanisms that were acquired during their initial colonization (Inoue et al. 2017) where early TEs innovated methods to evade silencing (Dawkins and Krebs 1979; Göke and Ng 2016). Once integrated into the genome, a subset of TEs would contain transcription factor binding sites already present in their own regulatory regions. This could initially provide a means to hijack critical host developmental programs to drive their own expression and delay their own extinction until mechanisms evolve to identify these elements as “non self” (Hermant and Torres‐Padilla 2021). As these insertions gradually become inert, they are eventually ignored by host TE surveillance mechanisms and are hypothesized to shift towards acquisition of gene regulatory function by the genome (Wells et al. 2023). This process opens the potential for TEs to be domesticated by the host in developmental or tissue‐specific patterns to the benefit of the host (Gerdes et al. 2016). Indeed, despite various epigenetic mechanisms that evolved to precisely silence these elements, a growing number of reports describe clear examples where TEs elicit positive and negative transcriptional effects on nearby genes (Rowe, Friedli, et al. 2013), and provide the framework of a potentially global gene regulatory system. In fact, retrotransposons were found to harbor over 20% of all transcription factor binding sites and up to 55% of specific transcription factor binding sites in both mice and humans (Table 1), with the LTR Class being the predominant retrotransposon suggested to be involved in these potential *cis*‐regulatory regions. This is consistent with the observation that LINE elements tend to degrade their own 5′ UTRs over time while recombination between the LTRs of ERVs results in relatively “intact” solitary LTRs, which harbor rich regulatory sequences. Interestingly, species‐specific transcription factor binding sites provided by retrotransposons were linked to species‐specific function, suggesting that their transposition drove unique gene regulatory innovations (Table 2). Such findings suggest that hosts risk compromised genome integrity in exchange for potential evolutionary catalysts for genetic innovation (Gerdes et al. 2016) often in a species‐specific manner.

The following three sections provide evidence of TEs functioning as *cis*‐regulatory DNA in human, mouse, and in disease contexts. These reports demonstrate that certain TEs are particularly adept at being repurposed for *cis‐*regulatory pathways in development, immune responses, aging, and various diseases. Recent findings support a model in which TEs predominantly function as *cis‐*regulatory enhancers as opposed to serving as silencers of nearby genes, which is either rare or perhaps more difficult to identify experimentally (Figure 2 and Table 2). Enhancers are DNA sequences that increase the transcription of associated genes, often by looping the DNA to bring transcription factors and other proteins into contact with the gene promoter (Andersson and Sandelin 2020). The presence of TEs in these enhancer regions can provide new or altered binding sites for transcription factors, thereby subtly altering gene expression patterns. This process can lead to the co‐evolution of new traits and adaptations, thus demonstrating the dynamic and influential role of TEs in genome evolution.

### 2.1 Evidence of TE DNA Activity in Human

The expansion of TEs that have *cis*‐regulatory potential has been linked to evolutionary changes in gene expression. The epigenetic reprogramming that occurs in early embryogenesis following fertilization (Figure 2) leads to the upregulation of multiple TEs. Pontis et al. showed that Krüppel‐associated box containing Zinc Finger Proteins regulate the activation of TE‐derived transcriptional *cis‐*regulators during epigenetic reprogramming. Activation of evolutionarily young TEs like Human Endogenous Retrovirus‐K (HERV‐K) and Human Endogenous Retrovirus‐H (HERV‐H) is thought to play a significant role in chromatin opening during human embryonic genome activation and act as enhancers in naïve human embryonic stem cells to drive host gene expression (Tables 1 and 2). In response, Krüppel‐associated box containing Zinc Finger Proteins of similar evolutionary age arose to repress the transcriptional activity of these TEs. Subsequent co‐evolution resulted in domestication of some Krüppel‐associated box containing Zinc Finger Proteins‐controlled TE‐based enhancers to serve as developmental and tissue‐specific enhancers (Pontis et al. 2019), further expanding the tissue and temporal regulatory options available to each host genome.

These expanded regulatory options are provided by TEs in the form of transcription factor binding sites that may have been deposited by TEs that originally harbored these binding sites when they were actively colonizing the genome. In many cases, TEs integrated in intergenic regions that are far away from genes and slowly eroded into fragments or fossils due to accumulating mutations. However, in some cases, they inserted within proximity of protein‐coding genes and, after the immediate threat to genome integrity was lost due to inactivating mutations, they either underwent the process of extinction or “exaptation”, which is an adaptation that fulfills a new function distinct from its originally selected function (Johnson 2019). Depending on the TE, their expansion could be in the hundreds to tens of thousands of nearly identical copies that are arrayed across genomes. These highly repetitive insertions deposited latent regulatory features across the genome and provide the framework for potentially coordinated gene regulatory networks specific to each TE family. To explore the functional potential of TEs as exapted *cis*‐regulatory units, an ape‐specific LTR of the HERV‐K class called LTR5HS was investigated in a pluripotent tumor cell line (Tables 1 and 2). LTR5HS normally activates during early pre‐implantation development and is present in about 700 copies in the human genome. When LTR5HS was targeted using Clustered Regularly Interspaced Short Palindromic Repeats (CRISPR) activation or CRISPR interference, this resulted in the reciprocal regulation of hundreds of human genes, suggesting direct effects (Figure 1). As both CRISPR activation and CRISPR interference operate on the manipulation of local chromatin, these observations could be side effects of the methodology. Therefore, Fuentes et al. carefully selected 6 distinct LTR5HS loci for deletion based on their potential to regulate nearby genes. All deletions showed significant downregulation of the nearby protein‐coding gene (Fuentes et al. 2018), suggesting that LTR5HS elements function as pervasive early embryonic enhancers in humans and apes.

To provide more physiological context, the concept of *cis*‐regulatory gene regulation was explored in the human germline, an epigenetic environment known to be conducive for TE expression. Primordial germ cells, precursors of sperm and egg, require temporal and precise epigenetic regulation for their correct differentiation. Specifically, primordial germ cells undergo extensive loss of DNA methylation, which is coincident with a rise in TE expression. This loss is essential to promote specification, and failure to do so leads to infertility. However, whether the increase in TE is needed is not clear (Gruhn et al. 2023). Transcription factor networks for primordial germ cell specification notably evolved quickly in mammals, complicating our understanding of human reproduction. Xiang et al. also reported on LTR5HS (Tables 1 and 2), which they found contain TE‐based enhancers that specifically aid in human primordial germ cell specification. LTR5HS TE‐based enhancers are activated and undergo epigenetic reprogramming that increases chromatin accessibility, DNA demethylation, histone 3 lysine 27 acetylation (H3K27ac) enrichment, and binding of key human primordial germ cell transcription factors, all of which are positively associated with increased gene expression. Inactivation of the TE‐based enhancers within LTR5HS using CRISPR inhibition significantly impacted germ cell specification, highlighting the essential role of TEs in human germ cell development (Xiang et al. 2022), and suggests that TE exaptation could be an underappreciated source of *cis*‐regulatory features in quickly evolving systems.

Another rapidly evolving system with clear signs of TE involvement is the mammalian placenta. This organ system demonstrates significant morphological and cellular diversity across species, which is thought to be driven by independent ERV acquisition events throughout evolution. These events span millions of years and provide species‐specific placental programs in a remarkable example of convergent evolution across all placental mammals, some marsupials, and at least one lizard (Cornelis et al. 2017; Imakawa et al. 2022). In placentas, TEs contribute to a wide variety of species‐specific gene regulatory programs in trophoblast cells by primarily acting as enhancers and promoters for tissue‐specific protein‐coding genes. Studies of TEs using epigenomic and gene expression data from primary human trophoblast and trophoblast stem‐cell lines identified over 10 ERV families that are strongly associated with interspecies gene expression differences by providing binding sites for transcription factors crucial for placental development. Genetic editing revealed that several TEs function as bona fide enhancers for key placental genes such as colony stimulating factor 1 receptor and pregnancy‐specific beta‐1‐glycoprotein 5 (Table 2). Additionally, in human trophoblast stem cell lines, TE‐derived enhancers are simultaneously enriched for both active and repressive histone marks. Upon differentiation into syncytiotrophoblast cells, these sites transition into active histone marks. Accordingly, syncytium formation was compromised when Mammalian‐wide interspersed repeat (MER) 50 elements were deleted near Major facilitator superfamily domain‐containing protein 2a and Tumor necrosis factor alpha‐induced protein 2, affecting gene expression through chromatin disruption and indicating a role for MER50 in trophoblast formation and function (Table 2) (Yu et al. 2023). Finally, a LTR10A element was found to directly regulate the expression and secretion of endoglin (Table 2), a gene with an important role in angiogenesis, leading to potential implications in a serious pregnancy complication that involves high blood pressure and other signs of organ damage called pre‐eclampsia (Frost et al. 2023). Collectively, these examples hint at significant contributions of TEs to human trophoblast gene regulation, suggesting that their activity may impact pregnancy outcomes and that their misregulation may be a novel and underappreciated source of fertility issues.

### 2.2 Evidence of TE DNA Activity in Mouse

In mice, one of the earliest and most famous reports of *cis‐*regulatory TEs driving gene regulation was described in 1994 when the 5′ LTR of an anti‐sense Intracisternal A‐Particle (IAP) was found to activate ectopic expression of the Agouti gene, leading to varying coat color, obesity, and diabetic‐like conditions (Table 2) (Michaud et al. 1994). Despite early indications of the importance of TEs such as IAP's control of the Agouti gene, the developmental and regulatory impact of TEs on normal biology has only recently begun to be elucidated.

Some mechanistic understanding into how TEs might function to control gene regulation came from studies researching Tripartite motif‐containing 28 (TRIM28), an essential epigenetic regulator thought to have roles in silencing ERVs to protect against disruption of early embryonic gene expression programs. In mouse Embryonic Stem Cells (ESCs), TRIM28 depletion leads to loss of Histone 3 Lysine 9 trimethylation (H3K9me3) and H3K27me3 repressive chromatin marks at certain ERVs, which are then replaced by the active enhancer chromatin mark H3K4me3. This leads to increased transcription of not only these ERVs but also nearby genes. These results could be confounded by TRIM28 also being involved in protein‐coding gene regulation; therefore, more direct evidence of ERV‐based *cis*‐regulatory control of nearby genes coming from careful measurements of TE and gene expression in early embryos is needed (Rowe, Kapopoulou, et al. 2013). Overall, ERV‐derived sequences were directly shown to either repress or enhance gene expression from adjacent promoters in transgenic embryos depending on the specific ERV and their sensitivity to TRIM28 manipulation.

To more clearly understand the scope and role of specific TE families in gene regulatory function, mouse ESCs and mouse trophoblast stem cells were used to survey TE function, as both cell types are known to be epigenetically permissive environments for TE expression. Chuong et al. compared enhancers in mouse and rat trophoblast stem cells and showed that species‐specific enhancers are highly enriched with ERVs (Chuong et al. 2013). Specifically, they found that the ERV family RLTR13D5 contributes hundreds of enhancers that interact with core trophoblast stem cell regulatory factors and can also drive gene expression when introduced into rat placental cells (Table 2). In a recent report, extensive analysis of transcriptomic and epigenomic data from mouse ESCs and mouse trophoblast stem cells was used to narrow down a list of potentially functional TEs (Figure 2). After integrative analysis combining TE and gene expression, along with details on chromatin accessibility, RLTR13D6 & RLTR9E in mouse ESCs and RLTR13B4 & RLTR13D5 elements in mouse trophoblast stem cells were identified as candidate sources of *cis‐*regulatory function (Figure 2 and Table 2) (Todd et al. 2019), with RLTR13D5 re‐identified, supporting the previous report (Chuong et al. 2013). After extensive testing and genome editing, two distinct TEs deleted in mouse trophoblast stem cells both demonstrated the anticipated regulatory effect on mitogen‐activated protein kinase 8 by RLTR13B4 and scavenger receptor class F member 2 by RLTR13D5. Conversely, only one out of four different TE deletions tested in mouse ESCs demonstrated clear enhancer activity on the nearby gene Tudor domain containing 12 by RLTR13D6 (Figure 2). Surprisingly, a separate lab showed that the expression of the previously tested A‐kinase anchoring protein 12 did indeed demonstrate significantly reduced expression after deletion of the exact same RLTR9E element, but in a different mouse ESC line (Figure 2 and Table 2) (Sundaram et al. 2017). These findings indicate that TEs offer complex and perhaps subtle roles in even closely related cellular and developmental contexts, where each TE family might operate under distinct *cis*‐regulatory mechanistic rules, rather than a generalizable mechanism.

To directly interrogate the gene regulatory capacity of a specific TE in live embryos and offer some insight into how TEs can regulate nearby genes, a systematic examination of mouse ERV‐L (MERV‐L) solitary LTRs was recently performed (Figure 2 and Table 2) (Yang et al. 2024). MT2_Mm is the LTR driving MERV‐L expression, in which MERV‐L has been directly linked to promoting totipotency in the murine 2‐cell stage embryos. It is thought to directly support zygotic genome activation through providing not only enhancer sequences for key 2‐cell genes but also functioning as 2‐cell specific promoters that contribute to transcript and isoform diversity (Table 2) (Honda et al. 2024; Modzelewski et al. 2021; Peaston et al. 2004). Using CRISPR inhibition, the expression of approximately 93% of MT2_Mm and 55% of the closely related MT2C_Mm insertions were downregulated in 2‐cell embryos (Figure 2 and Table 2). Notably, this perturbation resulted in the downregulation of hundreds of genes typically associated with zygotic genome activation, followed by embryonic arrest prior to blastocyst formation (Yang et al. 2024). This loss of viability was associated with drastic misregulation of putative MT2_Mm enhancer functions (Figure 2). Later, in mouse post‐implantation development, non‐canonical imprinting is mediated by ERV‐K LTRs. These LTRs act as imprinted promoters and help drive extra‐embryonic lineages (Figure 2 and Table 2) (Hanna et al. 2019). In these lineages, maternal H3K27me3 is replaced by DNA methylation during post‐implantation, while in the epiblast, it is silenced by bi‐allelic DNA methylation, highlighting the existence of different epigenetic regulation mechanisms fine‐tuned by the pervasive presence of TEs. These reports provide compelling evidence of the importance of TEs in early embryonic development *in vivo*, but a detailed mechanistic understanding of how TEs are regulated and how these same TEs regulate nearby genes is an ongoing investigation.

As in humans, rodent placental programs are highly influenced by TE enhancer function. The gene regulatory landscape of pan‐therian mammalian endometrial cells was directly investigated, and evidence for roughly 1500 endometrial‐expressed genes whose expression is exclusive to placental mammals was identified. This finding indicates a large‐scale rewiring of the gene regulatory network associated with the evolution of pregnancy (Lynch et al. 2011). Approximately 13% of these genes are found near the Eutherian‐specific TE MER20, which has enhancer, insulator, and repressor epigenetic signatures that are enriched for essential pregnancy‐associated transcription factors (Table 2). Furthermore, MER20 was demonstrated to regulate gene expression in response to progesterone and cAMP in endometrial cells, a rare example of direct regulation of TE expression through an external source. This study suggests that MER20 plays an ongoing and crucial role in developing a novel gene regulatory network for pregnancy across placental mammals.

Most identified TEs operating as *cis*‐regulatory elements play critical roles in early development. However, recent reports suggest that TEs play an increasing number of *cis*‐regulatory roles in immunological contexts. During evolution, regulatory networks driving innate immunity were under constant pressure to control pathogens, which themselves evolved to avoid the self or host immune system components. In studies of the mouse epigenomic response to type II interferon signaling, Horton et al. found that B2_Mm2, a subtype of SINE elements, contains STAT1 binding sites, thereby allowing these elements to act as interferon‐inducible enhancers (Table 2) (Horton et al. 2023). Furthermore, CRISPR deletion in mouse cells showed that a B2_Mm2 element enhances interferon‐inducible expression of *Dicer1*, a gene essential for post‐transcriptional gene regulation. Together, these findings support the ability of lineage‐specific TEs to drive the evolution of immune regulatory networks during the development of immune cell types (Horton et al. 2023). On a more global level, a comprehensive study revealed that expression from 84 TE subfamilies is overrepresented in a type of immune cell called Thymus cell (T‐cell) enhancers (Table 2), with ERVs enriched in accessible chromatin core domains bearing motifs for immune‐related transcription factors. Notably, SINEs are found in nucleosome‐containing boundaries (Table 2), which are often marked with the enhancer associated histone modification H3K4me1. TE‐rich enhancers are associated with genes related to lymphocyte and leukocyte biology and are shared across immune lineages. In support, immune‐specific enhancers are more TE‐rich, suggesting that TEs with beneficial motifs were frequently incorporated into gene networks as enhancers, accelerating the evolution of immune regulatory networks (Ye et al. 2020). While examples of TE *cis*‐regulatory function have only recently emerged from an immunological perspective, it suggests that other rapidly evolving biological systems subject to intense selective pressure will also be shaped by functional TE DNA elements and provide hints as to other biological contexts where TEs may be relevant.

### 2.3 Evidence of TE DNA Activity in Disease

As an organism ages, it slowly accumulates mutations and becomes sensitized to age‐related diseases, exhibiting an increased risk of mortality (Brunet and Berger 2014; Copley and Shorter 2023). Sensitivity to both aging and disease may be driven by a breakdown of epigenetic mechanisms, such as DNA hypomethylation, that are associated with the release of TEs from silencing, a phenomenon frequently observed in many cancers (Burns 2017). An early example of TE contribution to disease was a landmark 1988 study that showed loss of LINE‐1 silencing led to a *de novo* insertion into coagulation factor VII, leading to hemophilia A (Kazazian Jr et al. 1988). While striking, events like these were determined to be exceedingly rare, as comprehensive reviews of TE contribution to cancer revealed surprisingly few instances of novel TE insertions acting as driver mutations (Burns 2017). Recent advances in TE detection and investigation in developmental contexts provide some insight into more subtle ways in which TEs could potentially contribute to diseases beyond insertional mutagenesis. One possible contribution of TEs in disease etiology may be that TEs with *cis‐*regulatory potential are misregulated and subsequently alter nearby gene expression. Once the cell type in question has accumulated sufficient mutations or age‐related epigenetic inefficiencies, these TE‐based effects may begin to manifest. This concept has only recently been reported on, largely due to analytical and technological advances, such as long read sequencing technologies (Ewing et al. 2020), but provides an exciting new avenue of potential therapeutic targets that now include nearly 50% of the human genome.

Genetic and epigenetic changes that disrupt transcriptional networks are well‐established hallmarks of cancer (Esteller et al. 2024). In acute myeloid leukemia, a type of blood cancer in which bone marrow produces abnormal blood cells, a process called “onco‐exaptation” was shown to utilize the *cis*‐regulatory features of improperly reactivated TEs. Here, 6 ERV families demonstrating enhancer signatures were shown to drive nearby oncogene expression, potentially accelerating tumorigenesis (Table 2). In support, genetic editing and epigenetic silencing of these specific ERVs show that their deregulation affects adjacent gene expression. Furthermore, deletion or silencing of ERV‐derived enhancers reduces leukemia cell growth by inducing apoptosis (Deniz et al. 2020). Additionally, a recent report identified over 100 potential onco‐exaptation events across 4000 tumors spanning 15 cancer types. Further investigation of a normally hypermethylated SINE element, *AluJb*, revealed that it functioned as a cryptic promoter/enhancer for an RNA binding protein involved in gene regulation called Linear‐28b (known as LIN28B) in multiple lung cancer cell lines (Table 2). The authors showed that deleting this specific *AluJb* stopped oncogene expression, while DNA methylation‐controlled promoter activity demonstrated that TEs are necessary and sufficient for oncogene activation (Jang et al. 2019). The study highlighted the widespread impact of TE onco‐exaptation in cancer development and together, these reports revealed that ERVs are significant, previously overlooked sources of *cis*‐regulatory elements in acute myeloid leukemia and lung cancer that contribute to tumor heterogeneity and evolution.

To determine if direct targeting of TEs had therapeutic potential, Grillo et al. investigated the Tigger3a TE subfamily in prostate cancer cells (Table 2). Candidate Tigger3a elements were targeted using CRISPR inhibition, which when reduced led to decreased growth of two prostate cancer cell lines by 20%. Based on the overall genomic locations and proximity to affected genes, their findings supported a role for Tigger3a as a *cis*‐regulatory element downstream of the androgen receptor (Grillo et al. 2023). The critical finding that a subset of TEs can act as *cis*‐regulators in leukemia and prostate cancer cell lines provides evidence for a functional role in tumor cell progression and proliferation and highlights TEs as potential therapeutic targets in next‐generation strategies to treat these and other deadly cancers.

## 3 Transposons as a Source of Functional RNA

In addition to acting as *cis*‐regulatory DNA elements, TE transcripts can also regulate cellular processes and identity by acting as non‐coding RNAs (ncRNAs) and long non‐coding RNAs (lncRNAs) to regulate host cellular functions. TE RNAs can also play a more passive role by guiding alternative splicing patterns or contributing their own sequences into transcripts of protein‐coding genes in hybrid or “chimeric” transcripts. Recent surveys of the mouse and human genomes predicted nearly 30% of annotated TEs can be found within lncRNA transcripts (Topham et al. 2020) (Table 3). Given the universal phenomenon of robust retrotransposon reactivation during mammalian pre‐implantation embryogenesis, many studies have described the impact of TE RNA during embryonic development or embryonically derived cell lines, such as the maintenance of embryonic/induced pluripotent stem cells (iPSCs) and during later stages of development, as described below. Despite these recent efforts, our understanding of TE RNA functions in other cases where epigenetic breakdown occurs, such as during aging or disease, remains in its infancy.

### 3.1 Evidence of Functional TE RNA in Human

A prominent example of identifying and testing a functional TE‐derived RNA focused on HERV‐H, a primate‐specific TE family with approximately 231 insertions that are highly expressed in human ESCs (Table 3). Researchers targeted these 231 HERV‐H RNAs for depletion using short hairpin RNAs, resulting in dramatic changes to cellular morphology consistent with differentiation. Furthermore, pluripotency factors including octamer‐binding transcription factor 4 (OCT4), sex determining region Y box 2 (SOX2) and NANOG (Tír na nÓg–“Land of the Young” in the Irish language) were downregulated and differentiation markers GATA consensus Binding Protein 6 and Runt‐related transcription factor 1 were upregulated. Consistent with the role for HERV‐H in pluripotency, HERV‐H expression increased during reprogramming of fibroblasts into iPSCs, and its depletion decreased the number of iPSCs colonies generated. Immunoprecipitation assays to identify proteins associated with HERV‐H RNA revealed that these transcripts were largely associated with transcriptional coactivators important for pluripotency and iPSC generation. In the same report, depletion of HERV‐H had a commensurate suppressive impact on genes proximal to LTR7 insertions (the regulatory component of the HERV‐H family), suggesting that HERV‐H transcripts directly act as an RNA scaffold that recruits p300 and OCT4 to various LTR7 sites to induce the expression of adjacent genes known to regulate pluripotency programs (Lu et al. 2014). Further work on the requirement of HERV‐H during pre‐implantation development, and whether a similar mechanism exists in other species will be required to comprehensively assess the scope of this novel TE‐derived RNA function.

While the expression of TE transcripts is often upregulated in pluripotency, hybrid or “chimeric” lncRNA transcripts containing both mammalian gene and TE sequences have also been observed to be frequently driven by TE promoters. One example is human pluripotency‐associated transcripts 2, 3, and 5 that are expressed in inner cell mass (ICM) cells, human ESCs, and iPSCs. These genes are chimeric lncRNAs bearing up to 50% TE‐derived sequences, with the vast majority of the gene family (20/23) initiating transcription from distinct HERV‐H loci (Table 3). Specific knockdown of these lncRNAs in one blastomere of a human 2‐cell embryo led to its progeny failing to contribute to the ICM. Furthermore, knockdown of these lncRNAs inhibited reprogramming and reduced iPSC colony formation. More specifically, knockout of human pluripotency‐associated transcripts 5 led to increased expression of the let‐7 family of microRNAs, known regulators of pluripotency and reprogramming (Durruthy‐Durruthy et al. 2016). These findings support functional consequences for at least one TE‐derived lncRNA in the ICM and iPSCs, suggesting that this may just be the tip of the iceberg in TE‐driven impacts on pluripotency.

Beyond the early embryonic stages, a primate‐specific ERV from the MER41 family drives a lncRNA involved in fetal cardiomyocyte migration in human and non‐human primates (Table 3) (Wilson et al. 2020). Using human stem cell‐derived cardiomyocytes, hundreds of transcripts originating from MER41 insertions were identified. One MER41 insertion drove the expression of BRAF‐activated non‐coding RNA, a lncRNA that is exclusively expressed in primate fetal cardiomyocytes. In an *in vivo* model for heart development, cell migration was found to be altered in BRAF‐activated non‐coding RNA knockout, knockdown, and overexpression studies. Although not present in rodents, a BRAF‐activated non‐coding RNA knockin experiment in mice resulted in embryos with enlarged hearts compared to littermates, raising a potential but unexplored link between cell migration, organ size, and MER41.

While the previous studies support the role of TE‐derived non‐coding RNAs in controlling host function, TEs have also been demonstrated to affect non‐TE RNA by influencing splicing in a cell‐specific context. A humanized mouse model strategy was recently used to demonstrate how a human‐specific SINE element called *AluSx1* may have played a role in tail development among primates by altering splicing events (Figure 2 and Table 3) (Xia et al. 2024). Despite being one of the defining characteristics in various hominid lineages, the genetic mechanisms involved in tail loss are relatively unknown. This report demonstrated that the hominid specific insertion of an *AluSx1* into the intron of T‐box transcription factor T (*TBXT*) allows pairing with a neighboring but inverted *AluY* element that results in the creation of a hairpin loop in the primary *TBXT* transcript. This hairpin structure guided an alternative splicing event that resulted in a primate‐specific splicing isoform lacking exon 6 that directly altered the function of TBXT. To test whether the loss of exon 6 is associated with altered tail development, CRISPR/Cas9 was used to remove exon 6 from the *Tbxt* gene in mice, which resulted in severely shortened or missing tails. To mechanistically test whether the hairpin effect was responsible for tail loss, a humanized mouse model containing the primate *AluY* and *AluSx1* insertions was inserted into the corresponding mouse region as well as an engineered reverse complement sequence designed to also hairpin with an existing mouse sequence (Figure 2). While the humanized mouse failed to show a defect, likely due to failure of the mouse genome recognizing the primate sequences, the reverse complement sequence did produce shorter tails, with the extent of tail loss depending on the abundance of *Tbxt* transcript isoforms, thus further linking TBXT to tail development; however, whether TEs directly impact this process has yet to be determined. Although this example illustrates a more passive role for TEs, it demonstrates how cross‐species analyses involving humans, primates, and mice can uncover unique biological insights. These comparative approaches can reveal the functional consequences of TEs on gene regulation and assess their relevance across species.

### 3.2 Evidence of Functional TEs as RNA in Mouse

As in humans, TE RNAs are essential in mice, though their mechanisms of action are less clear. One example is the murine retrotransposon MERV‐L, which composes 3% of total RNA at the 2‐cell pre‐implantation embryo. The expression of MERV‐L is tightly regulated, in which reactivation is redundantly secured by two transcription factors, Double Homeobox X and oocyte‐specific homeobox 4, that recognize sequence motifs specific to MERV‐L (Guo et al. 2024; Sakashita et al. 2023). Mouse embryos that fail to transcribe MERV‐L elements after fertilization fail to develop beyond the 2‐cell stage (Table 3). MERV‐L knockdown experiments in pre‐implantation embryo RNAs resulted in embryonic arrest prior to blastocyst formation. Surprisingly, MERV‐L proteins were not essential for this effect, suggesting that either transcription of MERV‐L loci and/or functions of its RNA are required for development (Sakashita et al. 2023). Another essential TE in early embryos is LINE elements, which were shown to be essential for mouse development through the production of a nuclear RNA scaffold (Table 3). This structured transcript recruits TRIM28 and NUCLEOLIN to suppress Double Homeobox X activity, a key regulator of the 2‐cell transcriptional program (Percharde et al. 2018). Also in embryos, depletion of LINE‐1 protein while leaving LINE‐1 RNA intact resulted in impaired development, supporting a role for LINE‐1 RNA in exiting the 2‐cell stage, a necessary step toward the establishment of the pluripotent ICM. While the exact mechanisms behind TE transcript essentiality are often lacking, these and similar examples have identified distinct pathways and timing where TE transcripts impact and guide normal development in mice.

To gain some insight into possible generalizable roles for TE RNAs in early embryonic development, the lncRNA associated with Gene Expression and Transcription (*lincGET*) was the subject of thorough functional investigation (Figure 2 and Table 3). *LincGET* contains sequences from multiple ERV transposons such as MERV‐K and MERV‐L, which exhibit robust expression in mouse 2‐cell to 4‐cell embryos. Depletion of *LincGET* led to developmental arrest at the late 2‐cell stage (Figure 2). *LincGET* was determined to function as an inhibitor of exon skipping during this stage, affecting the *cis‐*regulatory capacities of the LTRs of MERV‐K and MERV‐L and splicing of genes (Wang et al. 2016). Later studies determined the expression of *LincGET* to be asymmetric and that it functions as a cell lineage regulator, with its expression biasing the fate of its daughter cells towards the ICM (Wang et al. 2018). Interestingly, this is one of the earliest detectable events involved in asymmetry observed in mouse pre‐implantation embryos.

Another attempt to uncover a generalizable role for TEs was through the rapid but reversible depletion of the heterochromatin adapter, TRIM28 in mouse ESCs, which resulted in the reactivation of its primary silencing targets, which are largely ERV class TEs (Asimi et al. 2022). While depletion of TRIM28 did not alter the expression of pluripotency markers, it was discovered that members of the ERV‐K family, and in particular the IAP family (Table 3), reactivate to form RNA transcript clusters that coalesce to form large non‐specific interactions that generate condensates or so‐called “phase separations” (Roden and Gladfelter 2021). Condensates are disordered regions that form membrane‐less compartments that can exclude molecules from entry due to features like charge or composition. In the case of the IAP RNA condensates, they were shown to specifically overlap with RNA Polymerase clusters. These IAP RNA‐based condensates were shown to form around and compete for enhancer regions, presumably displacing transcriptional machinery and leading to loss of pluripotency. While it is unclear whether IAP RNAs have normal biological roles in condensate formation or stability, the authors suggest that additional IAP transcripts from this class contribute to a process called “condensate hijacking”, that may contribute to the molecular basis of diseases where TRIM28 haploinsufficiency is observed.

Beyond the pre‐implantation embryo, a recent and remarkable example of convergent evolution involving an ERV TE was discovered to be essential for myelination in all jawed vertebrates, including mouse and human (Ghosh et al. 2024). Myelin is the insulating sheath surrounding axons in the central nervous system that allows for rapid transmission of nerve impulses to enable more complex brains and morphological diversity. Surprisingly, evidence suggests that multiple independent species‐specific acquisition events of RNLTR12‐like sequences from the ERV1 family of TEs generate the lncRNA “*RetroMyelin*” and are important for myelination during development. In rodents, knockdown of this lncRNA resulted in a drastic reduction of *in vivo* myelination. Functionally, *RetroMyelin* is a lncRNA that binds to the SOX10 transcription factor to regulate expression of myelin basic protein. To determine how conserved this SOX10 binding mechanism is, CRISPR/Cas9 was used to generate small deletions in both the fish *Danio rerio* and frog *Xenopus laevis* versions of *RetroMyelin*. The authors demonstrated a significant reduction of myelin in both species, suggesting a conserved role for this ERV‐derived RNA in regulating myelin in fish, amphibians, and mammals (Ghosh et al. 2024). Together, these data provide yet another example of the utility of cross‐species analysis to reveal the functional consequence of TEs on gene structure and function and provide further evidence that TE‐driven convergent evolution plays a central role in driving genetic innovation.

In addition to generating chimeric lncRNAs, as described above, TEs have also been observed to alter the regulation and function of protein‐coding genes by contributing to novel protein‐coding transcript isoforms (Figure 2 and Table 3). For example, TEs can function as alternative promoters for hundreds of genes during pre‐implantation development, resulting in alterations to regulatory regions in UTRs as well as truncations of the gene product when the TE is located within introns (Figure 2) (van de Lagemaat et al. 2003). Originally, Peaston et al. analyzed the transcriptome of mouse oocytes and early embryos and discovered transient alternative isoforms in which transcription was initiated from TEs, thus expanding the number of known gene isoforms and their functions (Peaston et al. 2004). As TEs are highly variable among species and have a unique relationship with host epigenetic regulatory machinery, specifically DNA methylation, these LTR‐initiated transcripts provide a source of regulatory variation and unique DNA methylation states between mouse, rat, and human oocytes (Brind'Amour et al. 2018). A recent cross‐species report spanning five different mammals looked at full‐length transcript information from embryos spanning mouse, pig, cow, rabbit, and rhesus monkey (Oomen et al. 2025). Nearly 20,000 TE‐driven genic transcripts were recovered, suggesting TE co‐option in early development is a common phenomenon. Interestingly, TEs displayed both shared and divergent regulation between species, suggesting both convergent and divergent regulatory patterns during mammalian development. One such chimeric transcript was shown to be essential in mouse pre‐implantation development. A TE‐derived N‐terminal truncated isoform of cyclin‐dependent kinase 2‐associated protein 1 or *Cdk2ap1* ^*ΔN(MT2B2)*^ is the major isoform present in mouse pre‐implantation embryos (Figure 2 and Table 3). *Cdk2ap1* ^*ΔN(MT2B2)*^ is essential for normal implantation of mouse embryos, as it promotes cell proliferation via direct interaction with the cell cycle regulator cyclin‐dependent kinase 2. In contrast, the canonical isoform, *Cdk2ap1* ^*CAN*^ impairs cell proliferation and causes embryonic lethality when overexpressed in mouse embryos. Interestingly, although *Cdk2ap1* ^*ΔN*^ is evolutionarily conserved, it is driven by distinct TE promoters in different mammals (Modzelewski et al. 2021). A second chimeric isoform in pre‐implantation mouse embryos was shown to be driven by the same TE (MT2B2) family but alters an unrelated gene, arginine N‐methyltransferase 6, or *Prmt6* ^*MT2B2*^(Figure 2 and Table 3). Here, the function of the chimeric protein also differs from that of its canonical isoform *Prmt6* ^*CAN*^. In overexpression studies in mouse embryos, *Prmt6* ^*MT2B2*^ mRNA promoted cell proliferation and differentiation when one blastomere from a 2‐cell embryo was injected with the chimeric transcript. The chimeric transcript supported blastomere development into epiblast cells at the blastocyst stage, while overexpression of *Prmt6* ^*CAN*^ repressed cell proliferation (Figure 2) (Honda et al. 2024). These examples suggest species‐specific transposon‐driven transcripts can yield both evolutionarily conserved or species‐specific alternative isoforms with potentially novel functions to govern essential biological divergence.

### 3.3 Evidence of Functional TE RNA in Disease

Relaxation of epigenetic regulatory networks is commonly observed in diseases, aging, and cancer, all of which are associated with disrupted transcriptional networks as well as reactivation of TEs (Esteller et al. 2024; Gorbunova et al. 2021; Mosaddeghi et al. 2023). Cancer cells have been described to exploit various regulatory pathways to promote malignancy, and TE transcripts provide an underappreciated source of proliferative and competitive advantages, as is the case of pluripotency‐associated HERV‐H transcripts in colorectal cancer (Table 3) (Yu et al. 2022). In two colorectal cancer cell lines and patient‐derived organoids, knockdown of HERV‐H resulted in impaired growth. Similar to the functional role of HERV‐H in human iPSCs, HERV‐H lncRNA binds to and colocalizes with coactivator Bromodomain Containing 4 foci in the nucleus of these cancer cells, subsequently altering downstream gene regulation. This first report of a novel HERV‐H‐Bromodomain Containing 4 regulatory axis provides evidence of cancer cells repurposing pluripotency programs to drive their own expansion and identifies potential targets for disease intervention.

In addition, TE activation can lead to the expression of novel lncRNAs that contribute to cancer. For example, in triple‐negative breast cancer, a solitary LTR from the LTR70 subfamily of ERV1 was linked to poor survival (Table 3) (Jin et al. 2019). Transcription of the lncRNA dubbed TROJAN begins transcription in an LTR70 locus and forms a chimeric lncRNA transcript with other ERVs and genomic sequences. TROJAN binds and promotes the degradation of the chromatin reader and metastatic repressor Zinc Finger MYND‐Type Containing 8. In triple‐negative breast cancer cell lines, TROJAN knockdown impaired proliferation, while overexpression increased it. *In vivo*, TROJAN downregulation reduced tumor volume in a mammary fat pad model in immunodeficient mice. Knockdown in lung, bone, and liver metastasis models led to fewer metastatic events, and deletion of TROJAN reduced tumor size and metastasis in triple‐negative breast cancer xenograft models. While it is unclear if lncRNAs involving other human ERVs can influence cancer progression, another well‐studied TE called HERV‐K has been observed and implicated in a variety of human diseases and cancers (Figure 3 and Table 3) (Costa and Vale 2023), suggesting that additional TE RNA‐based functions remain to be identified.

Chimeric TE transcripts that splice with protein‐coding genes have also been found to alter the expression and/or function of canonical transcripts, potentially driving cancer progression and worsening patient survival (Jang et al. 2019). Early examples demonstrated that dormant LTRs drive oncogene expression in Hodgkin's lymphoma (Babaian et al. 2016; Lamprecht et al. 2010). In one case, the THE1B LTR drove expression of Colony stimulating factor 1 receptor, resulting in a transcript with an extended 5′ UTR that is expressed in lymph nodes of Hodgkin's lymphoma patients but absent in healthy samples (Table 3) (Lamprecht et al. 2010). Similarly, the LTR, LOR1A, drove Interferon regulatory factor 5 expression, producing a novel transcript in Hodgkin's lymphoma but not in normal B‐cells (Table 3) (Babaian et al. 2016). In B‐cell lymphoma, an LTR2 insertion upstream of Fatty acid binding protein 7 creates a chimeric protein in which the canonical 24 amino acids are replaced with novel TE‐derived 20 amino acids at the N‐terminus (Table 3). Knockdown showed significant inhibition of cell growth, suggesting that the LTR2‐driven isoform is required for optimal cell proliferation (Lock et al. 2014). In lung squamous cell carcinoma, an HERV‐H LTR7 insertion drives a truncated Calbindin isoform lacking the N‐terminal 57 amino acids that is first detected in preinvasive lesions and associated with disease progression (Attig et al. 2023). Depletion of HERV‐H‐Calbindin in lung squamous cell carcinoma cells inhibits growth and induces senescence both *in vivo* and *in vivo* (Table 3). Interestingly, this isoform is also detected in early human embryos, specifically in the pluripotent epiblast, suggesting a link between early embryonic development and tumor progression (Singh et al. 2023). Altogether, these findings highlight the role of TE deregulation in malignant transformation through the formation of developmentally regulated, or perhaps cancer‐specific, chimeric transcripts. Interestingly, some of these chimeric transcripts may have important roles in early development but are later misregulated in cancer to the benefit of the tumor, offering a potential unique opportunity for novel therapeutic targets.

In addition to having roles in cancer, expression of TEs is highly tied to immune system function and dysfunction. One way this occurs is through promoting and strengthening anti‐viral responses via a TE‐derived lncRNA that has been exapted to sequester transcriptional repressive machinery. The lncRNA “ERV‐derived lncRNA positively regulates antiviral responses” (lnc‐EPAV) that is derived from a full‐length ERV1 RNA is robustly upregulated in bone‐marrow‐derived macrophages in response to viral infection (Zhou et al. 2019). Knockdown of lnc‐EPAV in macrophages infected with vesicular stomatitis virus greatly enhanced viral replication and titer (Table 3). Mechanistically, lnc‐EPAV was determined to be function through promoting the expression of v‐rel avian reticuloendotheliosis viral oncogene homolog A by sequestering its repressor Splicing factor, proline‐ and glutamine‐rich. To test the effect of lnc‐EPAV *in vivo*, a CRISPR mediated deletion of lnc‐EPAV was generated. As lnc‐EPAV heterozygous mice exhibited reduced growth, these mice were challenged with Vesicular stomatitis virus and found to have higher viral titers and reduced survival (Zhou et al. 2019). In another immune context, recent studies indicate that TEs can also generate smaller ncRNAs as substrates for innate pattern recognition receptors. These immunogenic ncRNAs are in part due to the various retrotransposition intermediates and virus‐like sequences (in the case of ERVs) which can result in “viral mimicry”, a phenomenon observed in aging, disease, and cancer various recent reviews on this topic can be found (Table 3) (Di Giorgio and Xodo 2022; Frost and Dubnau 2024; Lee, Ahmad, and Xu 2024; Ueda 2024). Viral mimicry can be largely explained by the unique ability of TEs to generate an abundant source ncRNAs spanning double‐stranded RNA (dsRNA), cDNA, and RNA:DNA hybrids. These RNA derived nucleic acids can be sensed by innate immune cells, although a comprehensive survey of immunogenic substrates produced from TEs and how they interact with the various sensors present in cells is still lacking. To this end, at least a few mechanisms have been proposed and demonstrated as to how TEs generate substrates for these RNA sensing pathways. Examples from both LINE‐1 and LTR retrotransposons (Figure 1) have been shown to engage in bi‐directional transcription from anti‐sense promoters (Dunn et al. 2006; Pinson et al. 2022). SINE elements are preferentially found in gene‐rich regions, in particular introns and 3′ UTRs (Table 3). This arrangement frequently generates dsRNA when inverted insertions are within proximity and transcribed, a process that is potentially regulated and involved in various biological processes in humans and primates (Lee, Ku, et al. 2024). These may suggest a generalized TE transcript domestication phenomenon shared across mammals.

Rather than contribute to disease, evidence for potential domestication of TEs to combat cancer comes from a recent report that may partially explain tumor resistance in the blind mole rat (Table 3). In blind mole rat tissues, expression of DNA MethylTransferase 1 is normally very low compared to humans and mice. As a result, in cases of hyperplasia and premalignancy, global levels of methylation decrease from baseline levels in blind mole rat cells, leading to the reactivation of TEs, specifically LINE transcripts. Blind mole rats appear to have co‐opted the generation of RNA:DNA hybrids, dsRNAs, and other immune sensing substrates to induce apoptosis of tumor cells through activation of the innate immune Cyclic GMP‐AMP synthase Stimulator of Interferon Genes sensor (Zhao et al. 2021). While xenograft experiments demonstrated that this pathway is present in both mice and humans, it is considerably more sensitive in blind mole rats.

The observation of TE‐derived RNA contributing to disease provides a unique opportunity and underappreciated source of therapeutic targets. Determining the various RNA‐based substrates generated from TEs and which sensors they preferentially activate holds promise in contributing to the identification of novel targets to treat cancer or alleviate various disease states. Indeed, the intentional reactivation of TEs using epigenetic inhibitors shows promise in “boosting” immune signaling in tumor cells, a phenomenon which can partially be explained by increased detection of dsRNAs (Grundy et al. 2022). This effect can be further enhanced using a combination of other epigenetic inhibitors that are associated with TE silencing and regulation. These strategies typically reactivate a spectrum of immunogenic and non‐immunogenic TEs but can also eventually disrupt normal gene regulation. Therefore, more careful surveys of specific reactivation events and examples such as the highly sensitive dsRNA sensing pathways in the blind mole rat are needed to fine‐tune and implement such strategies more broadly.

## 4 TEs as a Source of Functional Protein

The vast majority of TE insertions are truncated, mutated, or efficiently repressed (Pinson et al. 2022). In a study analyzing TEs across 19 mammalian species, only 0.05%–0.15% of insertions can produce proteins and are therefore capable of retrotransposition (Ueda et al. 2020). It is currently believed that only the youngest 80–100 human LINE element insertions can produce the proteins required for *de novo* insertions (Thawani et al. 2024). The basic Open Reading Frame (ORF) organization of LINE elements consists of two proteins (Figure 1), ORF1p (RNA‐binding) and ORF2p (endonuclease and reverse transcriptase, or reverse transcriptase function) (Denli et al. 2015). While no longer capable of retrotransposition in the human genome, ERVs, like their exogenous counterparts, largely consist of four coding domains (Figure 1) that are first translated as a polypeptide and then processed into individual proteins: (1) Group specific AntiGen “Gag” (structural polyprotein), (2) protease “Pro” (sometimes with dUTPase), (3) polyprotein “Pol” (reverse transcriptase, RNase, and integrase), and (4) Envelope “Env” (surface and transmembrane structural proteins) (Vargiu et al. 2016). Some TE‐derived proteins that have been domesticated retain similar activity to their ancestral versions, while others have been “fused” with nearby genes to create novel functions (Table 4). Some TE proteins have been exapted into essential functions in development and normal biology, while others have been hijacked by disease, but the full extent of TE protein co‐option is still emerging.

### 4.1 Evidence of Functional TE Protein in Mouse and Human Development

While many TEs are highly expressed and regulated in mammalian pre‐implantation embryos, there have been limited mechanistic studies to characterize potential functional roles (Grow et al. 2015; Modzelewski et al. 2021). This lack of information is primarily because of the scarcity of material and difficulty in identifying precise insertion coordinates, due to the highly repetitive nature of TEs in general. Nonetheless, the presence of TE protein expression is well‐established in early embryos. In humans, HERV‐K expression peaks at the 8‐cell and morula stages, with Gag forming viral‐like particles in the blastocyst (Table 4) (Grow et al. 2015). In mice, both MERV‐L RNA and Gag protein are highly abundant (Tables 3 and 4). MERV‐L Gag was recently implicated in modulating pluripotency factors OCT4 and SOX2 in the mouse pre‐implantation embryo during lineage specification. Mechanistically, MERV‐L Gag interacts with the Unconventional prefoldin RPB5 interactor protein, which was previously associated with pluripotency bias in mouse blastomeres. This interaction displaces the Unconventional prefoldin RPB5 interactor protein from binding to OCT4 and SOX2, causing their degradation (de la Rosa et al. 2024). However, antisense oligonucleotide‐mediated knockdown of MERV‐L Gag suggests it is the transcription of MERV‐L rather than the Gag protein that is essential for development (Table 4) (Sakashita et al. 2023), but targeting 100% of every version of Gag (coming from dozens of distinct locations) may be difficult or impossible. These examples underscore that the role of MERV‐L and other TE proteins is likely nuanced, and heterogeneous defects reflect the robust nature of early embryos. While the majority of TEs lack protein‐coding potential, individual insertions from both HERV‐K and MERV‐L TE families are predicted to have intact ORFs (Table 4); however, functions (if any), and whether they contribute to normal development have yet to be determined.

Some biological functions and mechanistic insights have been provided by studies in the placenta, a well‐known hotspot for TE protein expression, with the best‐known TE protein domestication involving syncytins (Table 4). As alluded to above, multiple acquisition events of ERV Env proteins independently occurred across all mammalian species, many marsupials, and at least one lizard and resulted in a remarkable example of convergent evolution of this organ system (Cornelis et al. 2017; Imakawa et al. 2022; Senft and Macfarlan 2021; Shimode 2023). First discovered in humans in the year 2000, Syncytin‐1 is derived from a repurposed HERV‐W locus in syncytiotrophoblasts that mediates cell–cell fusion (Table 4) (Blond et al. 2000). Likewise, syncytin‐2, derived from the Env of a HERV‐FRD insertion, is expressed in both the placenta and trophoblast cells to function in cell–cell fusion (Table 4) (Blaise et al. 2003; Esnault et al. 2008; Vargas et al. 2009). In contrast, SUPRESSYN, a protein derived from the Env from a HERV‐Fb1 insertion, opposes the syncytin function by binding the same receptor as syncytin‐1 to inhibit trophoblast syncytialization as well as cell–cell fusion (Table 4) (Sugimoto et al. 2019, 2013). SUPRESSYN appears to have been further repurposed for post‐implantation somatic development (Zhang et al. 2024). It is required in mesoderm and cardiomyocyte lineage specification during the differentiation of human pluripotent stem cells to cardiomyocytes, where it promotes degradation of secreted frizzled‐related proteins, resulting in altered WNT/β‐catenin signaling (Table 4). These examples showcase the incredible resourcefulness of the genome when developing and refining essential biological functions.

Outside of the embryo, recent efforts have focused on the roles of RT, Gag, and Env in mouse ESCs. Reverse transcriptase expression was linked to mouse ESC resistance to encephalomyocarditis and mouse hepatitis virus infection compared to mouse embryonic fibroblasts. When treated with reverse transcriptase inhibitors, mouse ESC viral titers and infection increase. These RTs generate viral complementary DNA which forms heteroduplexes with viral RNA, leading to the destruction of viral RNA and reduced viral infection (Table 4) (Wu et al. 2021). In human iPSCs, evidence suggests that Envs from HML‐2, a primate‐specific HERV‐K subtype, play a role in maintaining stemness where HML‐2 Envs localize to distinct compartments along the cell membrane (Table 4). Notably, knockdown of Env led to a reduction in iPSC colony formation and reduced expression of OCT4. Finally, HML‐2 Env directly binds to a protein called CD98 Heavy Chain, leading to activation of the mammalian Target Of Rapamycin and lysophosphatidylcholine acyltransferase 1 signaling pathways, whereas down regulation of HML‐2 Env resulted in differentiation into neuronal pathways (Table 4) (Wang et al. 2020). Whether the mechanisms that operate around TE‐derived proteins in human or mouse stem cells function similarly or identically to their *in vivo* embryonic counterparts is an exciting area of current investigation.

### 4.2 Evidence of Functional TE Protein in Disease

The relaxation of epigenetic surveillance mechanisms observed in early embryos during reprogramming shares similarities to the compromised epigenetics observed during disease progression such as cancer, neurodegeneration, as well as aging. Here, the common observation of widespread misregulation of TE proteins is observed, but how and to what degree they contribute to disease is unclear (Burns 2017; Gorbunova et al. 2021; Liang et al. 2024). Despite this, the detection of TE protein products has been used as a diagnostic marker for cancer cells with highly compromised epigenetic status for many years (Taylor et al. 2023). The impacts of the expression of these proteins are largely unknown; however, in specific instances, their presence is thought to worsen disease. For example, Li et al. revealed that Transactive response DNA binding protein 43 kDa (TDP‐43) mutations, which are major risk factors of Amyotrophic Lateral Sclerosis (ALS), suppress the activity of LINE1‐1 retrotransposition (Table 4) (Li et al. 2022). However, the causal relationship among TDP‐43 mutations, LINE‐1 retrotransposition, and ALS pathology is under debate. The N‐terminus of TDP‐43 directly interacts with LINE‐1 ORF1p, with the C‐terminus being essential for inhibiting LINE‐1 activity. Notably, ALS‐associated mutations in TDP‐43 are highly enriched in its C‐terminal domain. In addition, ORF1p is naturally abundant in the oocyte and pre‐implantation embryo, where TDP‐43 deficiency leads to a massive increase in LINE‐1 retrotransposition and severely impairs embryonic growth. Together, these data suggest a model in which ALS pathology is driven by cumulative LINE‐1 retrotransposition caused by TDP‐43 dysfunction over time, likely as a symptom but not the cause of ALS. As such, TDP‐43 appears to act as a guardian against LINE‐1 exposure during pre‐implantation embryogenesis, safeguarding genomic integrity, while its dysfunction later in life may contribute to LINE‐1 ORF1p‐driven disease etiology.

In early human embryos, HERV‐K is the most well established as a protein‐producing TE (Figure 3 and Table 4) (Grow et al. 2015). Thus, it is not surprising that HERV‐K proteins are the subject of most examples of TE involvement in disease (Figure 3). Whether there is a developmental role for HERV‐K remains unknown, but the Env protein has been implicated in various cancers (Figure 3) (Ko et al. 2021). Functional tests were conducted in a colorectal cancer cell line after CRISPR/Cas9 deletion of an HERV‐K locus on human chromosome 12. Knockout of this TE demonstrated reduced migration, invasion, and tumor colonization, while these features were enhanced in an overexpression model. Transcriptional analysis of the knockout cells showed a drastic decrease in Nuclear Protein 1 Transcriptional Regulator, an ER‐stress response factor (Figure 3). Validation through knockdown of this protein resulted in nearly identical phenotypes compared to the HERV‐K Env deletion cell line, suggesting a potential involvement for HERV‐K Env in this pathway. In addition, Np9, a small accessory protein that is translated from an alternative splice isoform of HERV‐K Env (Figure 3 and Table 4), has also been implicated in tumorigenesis (Chen et al. 2024). The original function of Np9 in the HERV‐K lifecycle is not clear, but Np9 is found in various normal tissues and is upregulated in specific tumor types (Fan and Qin 2024). Overexpression of Np9 resulted in DNA damage response *in vivo*, while stable knockdown of Np9 in a telomerase‐immortalized human umbilical vein endothelial cell line latently infected with Kaposi's sarcoma‐associated herpesvirus decreased the size of virally induced tumors and led to a drastic reduction of latency‐associated nuclear antigen expression *in vivo* (Chen et al. 2024). Together, these observations suggest a pathological role for multiple protein products from the HERV‐K Env gene in tumorigenesis, but whether HERV‐K is the major producer of TE proteins in development and disease is ongoing.

In addition to co‐opted functions, TE proteins can serve as a novel source of antigens in disease. A 2022 study used a machine‐learning approach to identify ERVs expressed across 29 solid tumor cancer cell types and further focused on ERVs that are associated with a cytotoxic T‐cell response (Figure 3 and Table 4). Six human leukocyte antigen epitopes from HERV‐K Gag and Pol that appeared to be expressed in cancers were able to prime CD8+ T‐cell clones to recognize and kill tumor cells expressing those epitopes (Figure 3 and Table 4) (Bonaventura et al. 2022). Furthermore, patients with hematological cancers exhibited higher levels of T‐cell reactivity to ERV peptides than do healthy controls (Table 4) (Saini et al. 2020). Epitopes from TEs may also drive autoimmunity, as levels of HERV‐K Env proteins are elevated, especially from the ERV‐K102 locus in systemic lupus erythematosus patient blood (Figure 3 and Table 4). Anti‐ERV‐K102 antibody levels in patient plasma enhanced neutrophil phagocytosis of ERV‐K102 Env protein through immune complex formation (Figure 3). Ultimately, this immunostimulatory ERV‐K Env protein leads to autoantibody production and can activate neutrophils, possibly contributing to inflammation present in systemic lupus erythematosus (Tokuyama et al. 2021). Thus, in diseases with elevated TE expression, TE peptides can serve as a source of epitopes, highlighting their potential for therapeutics.

Finally, in an example that blurs the lines between development and disease, extracellular HERV‐K Env was shown to not only serve as a biomarker for aging, but using cross‐species models, Liu et al. discovered a positive feedback loop between ERV reactivation and aging (Table 4) (Liu et al. 2023). Specifically, the accumulation of HERV‐K Env was associated with both cellular and tissue aging, and remarkably, this effect could be transmitted to young recipient cells that went on to display senescence phenotypes. This effect could be blocked by neutralizing antibodies against the HERV‐K Env protein. Notably, this overall mechanism appears to be present in species spanning mouse to primates, but interestingly, each species uses distinct ERVs to accomplish this convergent effect. Such exciting findings suggest that the inhibition of ERV‐mediated senescence could be a viable method to block tissue degeneration in vivo, suggesting possibilities for therapeutic strategies to alleviate age‐related diseases.

Despite recent discoveries of TE protein presence in disease, studies have only begun to directly address the therapeutic potential of TEs (Vergara Bermejo et al. 2020). A 2023 study found that in a KRAS‐driven lung cancer model with APOBEC3B expression model of lung adenocarcinoma, murine leukemia virus Env glycoproteins were the main target of anti‐tumor antibodies (Table 4). Treatment of KPAR‐challenged mice with an endogenous murine leukemia virus Env‐specific antibody increased survival. Furthermore, a proportion of human lung adenocarcinoma patients exhibit antibodies against HERV‐K Env, and increased levels of these antibodies following immune checkpoint blockade therapy are positively correlated with patient survival (Ng et al. 2023). Given the robust expression of ERV proteins in cancers, virus‐like vaccines consisting of Gag and Env proteins may protect against cancer cells expressing these proteins. In support, a Melanoma‐Associated RetroVirus virus‐like vaccine in which adenoviruses encoding the Melanoma‐Associated RetroVirus virus‐like proteins Gag and Env drive *in vivo* assembly of virus‐like particles displaying the cancer‐associated Melanoma‐Associated RetroVirus virus‐like Env protected against colorectal cancer growth and progression (Table 4) (Neukirch et al. 2019). Modifications to the immunosuppressive domain of Env in this virus‐like vaccine further increased T‐cell immunogenicity; in combination with anti‐programmed cell death protein‐1 treatment, it eradicated colorectal tumors and remarkably induced cross‐protection against triple‐negative breast cancer (Daradoumis et al. 2023). Altogether, the reactivation of TEs and associated proteins such as Env and Gag in cancer provides evidence for a promising strategy to specifically target certain cancer cells that could be potentially combined with current vaccine or immune targeting technologies.

## 5 Conclusion

Recent technological advances, including long‐read sequencing and other full‐transcript methodologies, have revolutionized our ability to study TEs. Additionally, the push for low‐input and single‐cell resolution in both transcriptomics and proteomics, coupled with the development of innovative analytical tools, has provided unprecedented insights into how TEs contribute to development and disease. The last few years have been especially exciting as a growing body of literature has uncovered some of the roles TEs have taken in contributing to normal biology as *cis‐*regulatory DNA elements, functional RNAs, and potentially domesticated TE protein products. Certain obstacles still need to be overcome before routine consideration of TEs across all areas of basic and biomedical research is commonplace. Most cases assembled here represent studies in which the role of TEs was not necessarily under consideration, but with an open mind and careful analysis, researchers brought the contributions of TEs to the forefront of these discoveries. As methods for identifying and manipulating individual TE insertions become more accessible to the broader research community, the resulting toolkit will enable a more TE‐centric approach for subsequent downstream functional validation. Recent advances in tool development open a multitude of possibilities in providing functional studies in TE gene regulatory networks (Fuentes et al. 2018). These recent efforts have provided functional validation of many fascinating examples of how TEs have influenced development, with ever‐increasing number of examples in development and disease. Equally promising will be unraveling how TEs can be effectively targeted in disease to alleviate or possibly reverse symptoms or the impacts of aging. For now, TEs stand poised to become the next frontier in decoding the intricate language of gene regulation, bridging the gap between development and disease while unlocking transformative possibilities for medicine and aging.

## Author Contributions

**Ten D. Li:** conceptualization (equal), data curation (equal), formal analysis (equal), investigation (equal), visualization (equal), writing – original draft (equal), writing – review and editing (equal). **Katelyn Toohill:** conceptualization (equal), data curation (equal), funding acquisition (equal), writing – original draft (equal), writing – review and editing (equal). **Andrew J. Modzelewski:** conceptualization (equal), data curation (equal), formal analysis (equal), funding acquisition (equal), investigation (equal), writing – original draft (equal), writing – review and editing (equal).

## Conflicts of Interest

The authors declare no conflicts of interest.

## Related WIREs Articles

Functional repeat‐derived RNAs often originate from retrotransposon‐propagated ncRNAs

RNA‐based regulation of transposon expression

SINEs

## Data Availability

Data sharing is not applicable to this article as no new data were created or analyzed in this study.
